# Phase Transitions in Transfer Learning for High-Dimensional Perceptrons

**DOI:** 10.3390/e23040400

**Published:** 2021-03-27

**Authors:** Oussama Dhifallah, Yue M. Lu

**Affiliations:** John A. Paulson School of Engineering and Applied Sciences, Harvard University, Cambridge, MA 02138, USA; yuelu@seas.harvard.edu

**Keywords:** transfer learning, statistics, phase transitions

## Abstract

Transfer learning seeks to improve the generalization performance of a target task by exploiting the knowledge learned from a related source task. Central questions include deciding what information one should transfer and when transfer can be beneficial. The latter question is related to the so-called negative transfer phenomenon, where the transferred source information actually reduces the generalization performance of the target task. This happens when the two tasks are sufficiently dissimilar. In this paper, we present a theoretical analysis of transfer learning by studying a pair of related perceptron learning tasks. Despite the simplicity of our model, it reproduces several key phenomena observed in practice. Specifically, our asymptotic analysis reveals a phase transition from negative transfer to positive transfer as the similarity of the two tasks moves past a well-defined threshold.

## 1. Introduction

Transfer learning [1,2,3,4,5] is a promising approach to improving the performance of machine learning tasks. It does so by exploiting the knowledge gained from a previously learned model, referred to as the source task, to improve the generalization performance of a related learning problem, referred to as the target task. One particular challenge in transfer learning is to avoid so-called negative transfer [6,7,8,9], where the transferred source information reduces the generalization performance of the target task. Recent literature [6,7,8,9] shows that negative transfer is closely related to the similarity between the source and target tasks. Transfer learning may hurt the generalization performance if the tasks are sufficiently dissimilar.

In this paper, we present a theoretical analysis of transfer learning by studying a pair of related perceptron learning tasks. Despite the simplicity of our model, it reproduces several key phenomena observed in practice. Specifically, the model reveals a sharp phase transition from negative transfer to positive transfer (i.e., when transfer becomes helpful) as a function of the model similarity.

### 1.1. Models and Learning Formulations

We start by describing the models for our theoretical study. We assume that the source task has a collection of training data ${\left\{\left(a_{s,i},y_{s,i}\right)\right\}}_{i=1}^{n_{s}}$, where $a_{s,i}\in R^{p}$ is the source feature vector and $y_{s,i}\in R$ denotes the label corresponding to $a_{s,i}$. Following the standard teacher–student paradigm, we assume that the labels ${\left\{y_{s,i}\right\}}_{i=1}^{n_{s}}$ are generated according to the following model:

(1) $$\begin{array}{c} y_{s,i}=\varphi \left(a_{s,i}^{⊤}\xi_{s}\right),\;\forall \;i\in \left\{1,\ldots ,n_{s}\right\}, \end{array}$$

where φ(·) is a scalar deterministic or probabilistic function and $\xi_{s}\in R^{p}$ is an unknown source teacher vector.

Similar to the source task, the target task has access to a different collection of training data ${\left\{\left(a_{t,i},y_{t,i}\right)\right\}}_{i=1}^{n_{t}}$, generated according to

(2) $$\begin{array}{c} y_{t,i}=\varphi \left(a_{t,i}^{⊤}\xi_{t}\right),\;\forall \;i\in \left\{1,\ldots ,n_{t}\right\}, \end{array}$$

where $\xi_{t}\in R^{p}$ is an unknown target teacher vector. We measure the similarity of the two tasks using

(3) $$\begin{array}{c} \rho \overset{\mathrm{def}}{=}\frac{\xi_{t}^{⊤}\xi_{s}}{∥\xi_{t}∥∥\xi_{s}∥}, \end{array}$$

with ρ=0 indicating two uncorrelated tasks whereas ρ=1 means that the tasks are perfectly aligned.

For the source task, we learn the optimal weight vector ${\hat{w}}_{s}$ by solving a convex optimization problem:

(4) $$\begin{array}{c} {\hat{w}}_{s}=\underset{w\in R^{p}}{\mathrm{argmin}}\;\frac{1}{p}\sum_{i=1}^{n_{s}}\ell \left(y_{s,i};a_{s,i}^{⊤}w\right)+\frac{\lambda }{2}{∥w∥}^{2}, \end{array}$$

where λ≥0 is a regularization parameter and ℓ(.;.) denotes some general loss function that can take one of the following two forms:

(5) $$\begin{array}{c} \left\{\begin{array}{cc} \ell \left(y;x\right)=\hat{\ell }\left(y-x\right), & \mathrm{for}\;\mathrm{regression}\;\mathrm{task}\\ \ell \left(y;x\right)=\hat{\ell }\left(yx\right), & \mathrm{for}\;\mathrm{classification}\;\mathrm{task}, \end{array}\right. \end{array}$$

where $\hat{\ell }\left(.\right)$ is a convex function.

In this paper, we consider a common strategy in transfer learning [4], which consists of transferring the optimal source vector, i.e., ${\hat{w}}_{s}$, to the target task. One popular approach is to fix a (random) subset of the target weights to values of the corresponding optimal weights learned during the source training process [10]. In our learning model, this amounts to the following target learning formulation:

(6) $$\begin{array}{cc} {\hat{w}}_{t}= & \underset{w\in R^{p}}{\mathrm{argmin}}\frac{1}{p}\sum_{i=1}^{n_{t}}\ell \left(y_{t,i};a_{t,i}^{⊤}w\right)+\frac{\lambda }{2}{∥w∥}^{2} \end{array}$$

(7) $$\begin{array}{cc} \; & \;\;\;s.t.\;\;Qw=Q{\hat{w}}_{s}. \end{array}$$

The vector ${\hat{w}}_{s}$ is the optimal solution of the source learning problem, and $Q\in R^{p\times p}$ is a diagonal matrix with diagonal entries drawn independently from a Bernoulli distribution with probability δ=m/p≤1. Here, *m* denotes the number of transferred components. Thus, on average, we retain δp number of entries from the source optimal vector ${\hat{w}}_{s}$. In addition to a possible improvement in the generalization performance, this approach can considerably lower the computational complexity of the target learning task by reducing the number of free optimization variables. In what follows, we refer to δ as the *transfer rate* and call (6) the *hard transfer* formulation.

Another popular approach in transfer learning is to search for target weight vectors in the vicinity of the optimal source weight vector ${\hat{w}}_{s}$. This can be achieved by adding a regularization term to the target formulation [11,12], which in our model becomes

(8) $${\hat{w}}_{t}=\underset{w\in R^{p}}{\mathrm{argmin}}\frac{1}{p}\sum_{i=1}^{n_{t}}\ell \left(y_{t,i};a_{t,i}^{⊤}w\right)+\frac{\lambda }{2}{∥w∥}^{2}+\frac{1}{2}{∥\Sigma \left(w-{\hat{w}}_{s}\right)∥}^{2},$$

with $\Sigma \in R^{p\times p}$ denoting some weighting matrix. In what follows, we refer to (8) as the *soft transfer* formulation, since it relaxes the strict equality in (6). In fact, the hard transfer in (6) is just a special case of the soft transfer formulation, if we set Σ to be a diagonal matrix in which the diagonal entries are either +∞ (with probability δ) or 0 (with probability 1−δ).

To measure the performance of the transfer learning methods, we use the generalization error of the target task. Given a new data sample $\left(a_{t,\mathrm{new}},y_{t,\mathrm{new}}\right)$ with $y_{t,\mathrm{new}}=\varphi \left(\xi_{t}^{⊤}a_{t,\mathrm{new}}\right)$, we assume that the target task predicts the corresponding label as

(9) $${\hat{y}}_{t,\mathrm{new}}=\hat{\varphi }\left[{\hat{w}}_{t}^{⊤}a_{t,\mathrm{new}}\right],$$

where $\hat{\varphi }\left(\cdot \right)$ is a predefined scalar function that might be different from φ(·). We then calculate the generalization error of the target task as

(10) $$\begin{array}{c} E_{\mathrm{test}}=\frac{1}{4^{\upsilon }}E\left[{\left(y_{t,\mathrm{new}}-\hat{\varphi }\left({\hat{w}}_{t}^{⊤}a_{t,\mathrm{new}}\right)\right)}^{2}\right], \end{array}$$

where the expectation is taken with respect to the new data $\left(a_{t,\mathrm{new}},y_{t,\mathrm{new}}\right)$. The variable υ allows us to write a more compact formula: υ is taken to be 0 for a regression problem and υ=1 for a binary classification problem. Finally, we use the training error

$$\begin{array}{c} E_{\mathrm{train}}=\frac{1}{p}\sum_{i=1}^{n_{t}}\ell \left(y_{t,i};a_{t,i}^{⊤}{\hat{w}}_{t}\right)+\frac{1}{2}{∥\Sigma \left({\hat{w}}_{t}-{\hat{w}}_{s}\right)∥}^{2}, \end{array}$$

to quantify the performance of the training process. Here, we measure the training error on the training data without regularization.

### 1.2. Main Contributions

The main contributions of this paper are two-fold, as summarized below:

#### 1.2.1. Precise Asymptotic Analysis

We present a precise asymptotic analysis of the transfer learning approaches introduced in (6) and (8) for Gaussian feature vectors and under regularity conditions on the eigenvalue distribution of the weighting matrix Σ. Specifically, we show that, as the dimensions $p,n_{s},n_{t}$ grow to infinity with the ratios $\alpha_{s}=n_{s}/p,\alpha_{t}=n_{t}/p$ fixed, the generalization errors of the hard and soft formulations can be exactly characterized by the solutions of two low-dimensional *deterministic* optimization problems. (See Theorem 1 and Corollary 1 for details.) Our asymptotic predictions hold for any convex loss functions used in the training process, including the squared loss for regression problems and logistic loss commonly used for binary classification problems.

**Figure 1:**
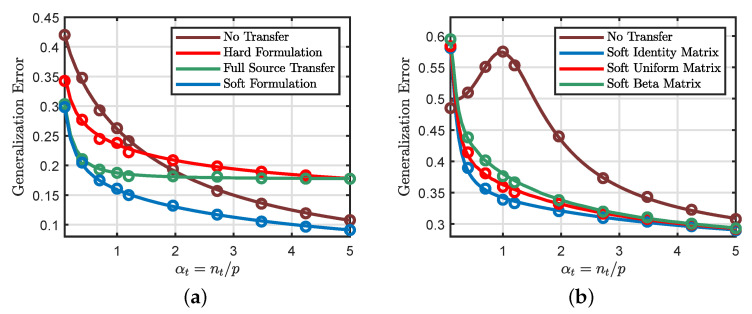
Theoretical predictions v.s. numerical simulations obtained by averaging over 100 independent Monte Carlo trials with dimension p=2500. (**a**) Binary classification with logistic loss. We take $\alpha_{s}=10\alpha_{t}$, λ=0.3, $\Sigma =I_{p}/\sqrt{5}$, and ρ=0.85, where $\alpha_{s}=n_{s}/p$ and $\alpha_{t}=n_{t}/p$. The functions φ(·) and $\hat{\varphi }\left(\cdot \right)$ are both the sign function. For hard transfer, we set the transfer rate to be δ=0.5. *Full source transfer* corresponds to δ=1.0, whereas *no transfer* corresponds to δ=0. (**b**) Nonlinear regression using quadratic loss, where φ(·) is the ReLu function and $\hat{\varphi }\left(\cdot \right)$ is the identity function. Soft identity, beta, and uniform matrices refer to different choices of the weighting matrix in (8). Soft Identity Matrix: Σ is an identity matrix. Soft Uniform Matrix: Σ is a random matrix with diagonal elements drawn from the uniform distribution. Soft Beta Matrix: Σ is a random matrix with diagonal elements drawn from the beta distribution. We scale all diagonal elements of Σ to have the same mean. We also take $\alpha_{s}=10\alpha_{t}$, λ=0.1, and ρ=0.8.

As illustrated in Figure 1, our theoretical predictions (drawn as solid lines in the figures) reach excellent agreement with the actual performance (shown as circles) of the transfer learning problem. Figure 1a considers a binary classification setting with logistic loss, and we plot the generalization errors of different transfer approaches as a function of the target data/dimension ratio $\alpha_{t}=n_{t}/p$. We can see that the hard transfer formulation (6) is only useful when $\alpha_{t}$ is small. In fact, we encounter negative transfer (i.e., hard transfer performing worse than no transfer) when $\alpha_{t}$ becomes sufficiently large. Moreover, the soft transfer formulation (8) seems to achieve more favorable generalization errors compared to the hard formulation. In Figure 1b, we consider a regression setting with a squared loss and explore the impact of different weighting schemes on the performance of the soft formulation. We can see that the soft formulation indeed considerably improves the generalization performance of the standard learning method (i.e., learning the target task without any knowledge transfer).

#### 1.2.2. Phase Transitions

Our asymptotic characterizations reveal a phase transition phenomenon in the hard transfer formulation. Let

$$\delta^{★}=\underset{0\leq \delta \leq 1}{\mathrm{argmin}}\;E_{\mathrm{test}}\left(\delta \right),$$

be the optimal transfer rate that minimizes the generalization error of the target task. Clearly, $\delta^{★}=0$ corresponds to the negative transfer regime, where transferring the knowledge of the source task will actually hurt the performance of the target task. In contract, $\delta^{★}>0$ signifies that we have entered the positive transfer regime, where transfer becomes helpful.

**Figure 2:**
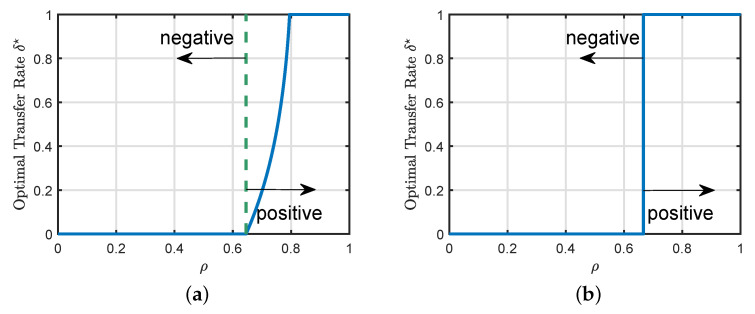
Phase transitions of the hard transfer formulation. When the similarity ρ between the two tasks is small, we are in the negative transfer regime, where we should not transfer the knowledge from the source task. However, as ρ moves past a critical threshold, we enter the positive transfer regime. (**a**) Binary classification with squared loss, with parameters $\alpha_{t}=2$, $\alpha_{s}=2\alpha_{t}$, and λ=0. Both φ(·) and $\hat{\varphi }\left(\cdot \right)$ are the sign function. (**b**) Nonlinear regression with squared loss, with parameters $\alpha_{t}=2$, $\alpha_{s}=2\alpha_{t}$, and λ=0. φ(.) is the ReLu function and $\hat{\varphi }\left(.\right)$ is the identity function.

Figure 2a illustrates the phase transition from negative to positive transfer regimes in a binary classification setting, as the similarity ρ between the two tasks moves past a critical threshold. Similar phase transition phenomena also appear in nonlinear regression, as shown in Figure 2b. Interestingly, for this setting, the optimal transfer rate *jumps* from $\delta^{★}=0$ to $\delta^{★}=1$ at the transition threshold.

For general loss functions, the exact locations of the phase transitions can only be found numerically by solving the deterministic optimization problems in our asymptotic characterizations. For the special case of squared loss with no regularization, however, we are able to obtain the following simple analytical characterization for the phase transition threshold: We are in the positive transfer regime *if and only if*

(11) $$\rho >\rho_{c}\left(\alpha_{s},\alpha_{t}\right)=1-\frac{E\left[\varphi^{2}\left(z\right)\right]-E^{2}\left[z\varphi \left(z\right)\right]}{2\;E^{2}\left[z\varphi \left(z\right)\right]}\left(\frac{1}{\alpha_{t}-1}-\frac{1}{\alpha_{s}-1}\right),$$

where *z* is a standard Gaussian random variable. This result is shown in Proposition 1.

By the Cauchy–Schwarz inequality, $E\left[\varphi^{2}\left(z\right)\right]\geq E^{2}\left[z\varphi \left(z\right)\right]$. It follows that $\rho_{c}\left(\alpha_{s},\alpha_{t}\right)$ is an increasing function of $\alpha_{t}$ and a decreasing function of $\alpha_{s}$. This property is consistent with our intuition: As we increase $\alpha_{t}$, the target task has more training data to work with, and thus, we should set a higher bar in terms of when to transfer knowledge. As we increase $\alpha_{s}$, the quality of the optimal source vector becomes better, in which case, we can start the transfer at a lower similarity level. In particular, when $\alpha_{t}>\alpha_{s}$, we have $\rho_{c}\left(\alpha_{s},\alpha_{t}\right)>1$ and, thus, the inequality in (11) is never satisfied (because |ρ|≤1 by definition). This indicates that no transfer should be done when the target task has more training data than the source task.

### 1.3. Related Work

The idea of transferring informaton between different domains or different tasks was first proposed in [1] and further developed in [2]. It has been attracting significant interest in recent literature [4,5,6,7,8,9,11,12]. While most work focuses on the practical aspects of transfer learning, there have been several studies (e.g., [13,14]) that seek to provide analytical understandings of transfer learning in simplified models. Our work is particularly related to [14], which considers a transfer learning model similar to ours but for the special case of linear regression. The analysis in this paper is more general as it considers arbitrary convex loss functions. We would also like to mention an interesting recent work that studies a different but related setting referred to as knowledge distillation [15].

In term of technical tools, our asymptotic predictions are derived using the convex Gaussian min–max theorem (CGMT). The CGMT was first introduced in [16] and further developed in [17]. It extends a Gaussian comparison inequality first introduced in [18]. It particularly uses convexity properties to show the equivalence between two Gaussian processes. The CGMT has been successfully used to analyze convex regression formulations [17,19,20] and convex classification formulations [21,22,23,24].

### 1.4. Organization

The rest of this paper is organized as follows. Section 2 states the technical assumptions under which our results are obtained. Section 3 provides an asymptotic characterization of the soft transfer formulation. Precise analysis of the hard transfer formulation is presented in Section 4. We provide remarks about our approach in Section 5. Our theoretical predictions hold for general convex loss functions. We specialize these results to the settings of nonlinear regression and binary classification in Section 6, where we also provide additional numerical results to validate our predictions. Section 7 provides detailed proof of the technical statements introduced in Section 3 and Section 4. Section 8 concludes the paper. The Appendix provides additional technical details.

## 2. Technical Assumptions

The theoretical analysis of this paper is carried out under the following assumptions.

**Assumption** **1** (Gaussian Feature Vectors)**.** *The feature vectors ${\left\{a_{s,i}\right\}}_{i=1}^{n_{s}}$ and ${\left\{a_{t,i}\right\}}_{i=1}^{n_{t}}$ are drawn independently from a standard Gaussian distribution. The vector $\xi_{s}\in R^{p}$ can be expressed as $\xi_{s}=\rho \xi_{t}+\sqrt{1-\rho^{2}}\xi_{r}$, where the vectors $\xi_{t}\in R^{p}$ and $\xi_{r}\in R^{p}$ are independent from the feature vectors, and they are generated independently from a uniform distribution on the unit sphere.*

Moreover, our results are valid in a high-dimensional asymptotic setting, where the dimensions *p*, $n_{s}$, $n_{t}$, and *m* grow to infinity at fixed ratios.

**Assumption** **2** (High-dimensional Asymptotic)**.** *The number of samples and the number of transferred components in hard transfer satisfy $n_{s}=n_{s}\left(p\right)$, $n_{t}=n_{t}\left(p\right)$, and m=m(p), with $\alpha_{s,p}=n_{s}\left(p\right)/p\to \alpha_{s}>0$, $\alpha_{t,p}=n_{t}\left(p\right)/p\to \alpha_{t}>0$, and $\delta_{p}=m\left(p\right)/p\to \delta >0$ as p→∞.*

The CGMT framework makes specific assumptions about the loss function and the feasibility sets. To guarantee these assumptions, this paper considers a family of loss functions that satisfy the following conditions. Note that the assumption is stated for the target task, but we assume that it is also valid for the source task.

**Assumption** **3** (Loss Function)**.** *If λ>0, the loss function ℓ(y;.) defined in *(5)* is a proper convex function in R. If λ=0, the loss function ℓ(y;.) defined in *(5)* is a proper strongly convex function in R, where the constant S>0 is a strong convexity parameter. In this case, we only consider the case when $\alpha_{t}>1$. Define a random function $L\left(x\right)=\sum_{i=1}^{n_{t}}\ell \left(y_{i};x_{i}\right)$, where $y_{i}∼\varphi \left(z_{i}\right)$, with $\left\{z_{i}\right\}$ being a collection of independent standard normal random variables and ∼ denoting equality in distribution. Denote by ∂L the sub-differential set of L(x). Then, for any constant C>0, there exists a constant R>0 such that*

(12) $$\begin{array}{c} \left\{\begin{array}{c} P\left(\underset{∥v∥\leq C\sqrt{n_{t}}}{\sup}\;\underset{s\in \partial L(v)}{\sup}∥s∥\leq R\sqrt{n_{t}}\right)\overset{p\to \infty }{\to }1.\\ P\left(\underset{∥v∥\leq C\sqrt{n_{t}}}{\sup}\left|L(v)\right|\leq Rn_{t}\right)\overset{p\to \infty }{\to }1. \end{array}\right. \end{array}$$

Furthermore, we consider the following assumption to guarantee that the generalization error defined in (10) concentrates in the large system limit.

**Assumption** **4** (Regularity Conditions)**.** *The data-generating function φ(·) is independent from the feature vectors. Moreover, the following conditions are satisfied.*

- *φ(·) and $\hat{\varphi }\left(\cdot \right)$ are continuous almost everywhere in R. For every h>0 and z∼N(0,h), we have $0<E[\varphi^{2}\left(z\right)]<+\infty$ and $0<E[{\hat{\varphi }}^{2}\left(z\right)]<+\infty$.*
- *For any compact interval [c,C], there exists a function g(·) such that*
  $$\underset{h\in [c,C]}{\sup}{\left|\hat{\varphi }\left(hx\right)\right|}^{2}\leq g\left(x\right)\;\mathrm{for}\;\mathrm{all}\;x\in R.$$
  *Additionally, the function g(·) satisfies $E[g^{2}\left(z\right)]<+\infty$, where z∼N(0,1).*

Finally, we introduce the following assumption to guarantee that the training and generalization errors of the soft formulation can be asymptotically characterized by deterministic optimization problems.

**Assumption** **5** (Weighting Matrix)**.** *Let $\Lambda =\Sigma^{⊤}\Sigma$, where* **Σ** *is the weighting matrix in the soft transfer formulation. Let $\sigma_{\min,1}\left(\Lambda \right)$ and $\sigma_{\min,2}\left(\Lambda \right)$ denote its two smallest eigenvalues. There exists a constant $\mu_{\min}\geq 0$ such that*

(13) $$\begin{array}{c} \left\{\begin{array}{c} \sigma_{\min,1}\left(\Lambda \right)\overset{\;\;p\;\;}{\to }\mu_{\min}\\ |\sigma_{\min,1}\left(\Lambda \right)-\sigma_{\min,2}\left(\Lambda \right)|\overset{\;\;p\;\;}{\to }0. \end{array}\right. \end{array}$$

*Moreover, we assume that empirical distribution of the eigenvalues of the matrix* **Λ** *converges weakly to a probability distribution $P_{\mu }\left(\cdot \right)$.*

The above assumptions are essential to show that the soft formulation in (8) concentrates in the large system limit. We provide more details about these assumptions in Appendix A.

## 3. Sharp Asymptotic Analysis of Soft Transfer Formulation

In this section, we study the asymptotic properties of soft transfer formulation. Specifically, we provide a precise characterization of the training and generalization errors corresponding to (8).

The asymptotic performance of the source formulation defined in (4) has been studied in the literature [24]. In particular, it has been shown that the asymptotic limit of the source formulation in (4) can be quantified by the following deterministic optimization problem:

(14) $$\min_{\begin{array}{c} q_{s},r_{s}\geq 0 \end{array}}\underset{\begin{array}{c} \sigma_{s}>0 \end{array}}{\sup}\;\alpha_{s}E\left[M_{\ell (Y_{s},.)}\left(r_{s}H_{s}+q_{s}S_{s};\frac{r_{s}}{\sigma_{s}}\right)\right]-\frac{r_{s}\sigma_{s}}{2}+\frac{\lambda }{2}\left(q_{s}^{2}+r_{s}^{2}\right),$$

where $Y_{s}=\varphi \left(S_{s}\right)$ and $H_{s}$ and $S_{s}$ are two independent standard Gaussian random variables. Furthermore, the function $M_{\ell (Y_{s},.)}$ introduced in the scalar optimization problem (14) is the Moreau envelope function defined as

(15) $$\begin{array}{c} M_{\ell (y,.)}\left(a;b\right)=\min_{c\in R}\ell \left(y;c\right)+\frac{1}{2b}{\left(c-a\right)}^{2}. \end{array}$$

The expectation in (14) is taken over the random variables $H_{s}$ and $S_{s}$.

In our work, we focus on the target problem with soft transfer, as formulated in (8). It turns out that the asymptotic performance of the target problem can also be characterized by a deterministic optimization problem:

(16) $$\begin{array}{cc} \min_{\begin{array}{c} q_{t},r_{t}\geq 0 \end{array}} & \underset{\sigma_{t}>-\mu_{\min}}{\sup}-\frac{\sigma_{t}r_{t}^{2}}{2}+\frac{1}{2}\left(\left(1-\rho^{2}\right){\left(q_{s}^{★}\right)}^{2}+{\left(r_{s}^{★}\right)}^{2}\right)T_{2}\left(\sigma_{t}\right)\\ \; & \;+\alpha_{t}E\left[M_{\ell (Y_{t},.)}\left(r_{t}H_{t}+q_{t}S_{t};T_{1}\left(\sigma_{t}\right)\right)\right]+\frac{\lambda }{2}\left(q_{t}^{2}+r_{t}^{2}\right)\\ \; & \;-\frac{1}{2}{\left(q_{t}-\rho q_{s}^{★}\right)}^{2}\left(\sigma_{t}-1/T_{1}\left(\sigma_{t}\right)\right), \end{array}$$

where $Y_{t}=\varphi \left(S_{t}\right)$, and $H_{t}$ and $S_{t}$ are independent standard Gaussian random variables. Additionally, $\mu_{\min}$ represents the minimum value of the random variable with distribution $P_{\mu }\left(.\right)$ as defined in Assumption 5. In the formulation (16), the constants $q_{s}^{★}$ and $r_{s}^{★}$ are optimal solutions of the asymptotic formulation given in (14). Moreover, the functions $T_{1}\left(.\right)$ and $T_{2}\left(.\right)$ are defined as follows:

$$\begin{array}{c} T_{1}\left(\sigma_{t}\right)=E_{\mu }\left[1/\left(\mu +\sigma_{t}\right)\right],\;T_{2}\left(\sigma_{t}\right)=E_{\mu }\left[\mu \sigma_{t}/\left(\mu +\sigma_{t}\right)\right], \end{array}$$

where the expectations are taken over the probability distribution $P_{\mu }\left(.\right)$ defined in Assumption 5.

**Theorem** **1** (Precise Analysis of the Soft Transfer)**.** *Suppose that Assumptions 1–5 are satisfied. Then, the training error corresponding to the soft transfer formulation in *(8)* converges in probability as follows:*

(17) $$\begin{array}{c} E_{\mathrm{train}}\overset{p\to \infty }{\to }C_{t}^{★}-\frac{\lambda }{2}\left({\left(q_{t}^{★}\right)}^{2}+{\left(r_{t}^{★}\right)}^{2}\right), \end{array}$$

*where $C_{t}^{★}$ denotes the minimum value achieved by the scalar formulation introduced in *(16)*, and $q_{t}^{★}$ and $r_{t}^{★}$ are optimal solutions of the scalar formulation in *(16)*. Moreover, the generalization error introduced in *(10)* corresponding to soft transfer formulation converges in probability as follows:*

(18) $$\begin{array}{c} E_{\mathrm{test}}\overset{p\to \infty }{\to }\frac{1}{4^{\upsilon }}E\left[{\left(\varphi \left(\nu_{1}\right)-\hat{\varphi }\left(\nu_{2}\right)\right)}^{2}\right], \end{array}$$

*where $\nu_{1}$ and $\nu_{2}$ are two jointly Gaussian random variables with zero mean and a covariance matrix given by*

$$\begin{array}{c} \left[\begin{array}{cc} 1 & q_{t}^{★}\\ q_{t}^{★} & {\left(q_{t}^{★}\right)}^{2}+{\left(r_{t}^{★}\right)}^{2} \end{array}\right]. \end{array}$$

The proof of Theorem 1 is based on the CGMT framework [17] (Theorem 6.1). A detailed proof is provided in Section 7.3. The statements in Theorem 1 are valid for a general convex loss function and general learning models that can be expressed as in (1) and (2). The analysis in Section 7.3 shows that the deterministic problems in (14) and (16) are the asymptotic limits of the source and target formulations given in (4) and (8), respectively. Moreover, it shows that the deterministic problems (14) and (16) are strictly convex in the minimization variables. This implies the uniqueness of the optimal solutions of the minimization problems.

**Remark** **1.** *The results of the theorem show that the training and generalization errors corresponding to soft transfer formulation can be fully characterized using the optimal solutions of scalar formulation in *(16)*. Moreover, from its definition, *(16)* depends on the optimal solutions of the scalar formulation in *(14)* of the source task. This shows that the precise asymptotic performance of the soft transfer formulation can be characterized after solving two scalar deterministic problems.*

## 4. Sharp Asymptotic Analysis of Hard Transfer Formulation

In this section, we study the asymptotic properties of hard transfer formulation. We then use these predictions to rigorously prove the existence of phase transitions from negative to positive transfer.

### 4.1. Asymptotic Predictions

As mentioned earlier, the hard transfer formulation can be recovered from (8) as a special case where the eigenvalues of the matrix Λ are +∞ with probability δ and 0 otherwise. Thus, we obtain the following result as a simple consequence of Theorem 1.

**Corollary** **1.** *Suppose that Assumptions 1–4 are satisfied. Then, the asymptotic limit of the hard formulation defined in *(6)* is given by the following deterministic formulation:*

(19) $$\begin{array}{cc} \min_{\begin{array}{c} q_{t},r_{t}\geq 0 \end{array}}\underset{\sigma >0}{\sup}\; & \frac{\lambda }{2}\left(q_{t}^{2}+r_{t}^{2}\right)+\frac{\sigma \delta }{2}\left[\left(1-\rho^{2}\right){\left(q_{s}^{★}\right)}^{2}+{\left(r_{s}^{★}\right)}^{2}\right]\\ \; & \;+\alpha_{t}E\left[M_{\ell (Y_{t},.)}\left(r_{t}H_{t}+q_{t}S_{t};\frac{1-\delta }{\sigma }\right)\right]-\frac{\sigma r_{t}^{2}}{2}\\ \; & \;+\frac{\sigma \delta }{2(1-\delta )}{\left(q_{t}-\rho q_{s}^{★}\right)}^{2}. \end{array}$$

*Additionally, the training and generalization errors associated with the hard formulation converge in probability to the limits given in *(17)* and *(18)*, respectively.*

### 4.2. Phase Transitions

As illustrated in Figure 2, there is a phase transition phenomenon in the hard transfer formulation, where the problem moves from negative transfer to positive transfer as the similarity of the source and target tasks increases. For general loss functions, the exact location of the phase transition boundary can only be determined by numerically solving the scalar optimization problem in (19).

For the special case of squared loss, however, we are able to obtain analytical expressions. For the rest of this section, we restrict our discussions to the following special settings:

(a) The loss function ℓ(·,·) in (4) and (6) is the squared loss, i.e., $\ell \left(y,x\right)=\frac{1}{2}{\left(y-x\right)}^{2}$.
(b) The regularization strength λ=0 in the source and target formulations (4) and (6).
(c) The data/dimension ratios $\alpha_{s}$ and $\alpha_{t}$ satisfy $\alpha_{s}>1$ and $\alpha_{t}>1$.

We first consider a nonlinear regression task, where the function φ(·) in the generative models (1) and (2) can be arbitrary and where the function $\hat{\varphi }\left(\cdot \right)$ in (9) is the identity function.

**Proposition** **1** (Regression Phase Transition)**.** *In addition to conditions (a)–(c) introduced above, assume that the predefined function $\hat{\varphi }\left(\cdot \right)$ in *(9)* is the identity function. Let $\delta^{★}$ be the optimal transfer rate that leads to the lowest generalization error in the hard formulation *(6)*. Then,*

(20) $$\begin{array}{c} \delta^{★}=\left\{\begin{array}{cc} 0 & \mathrm{if}\;\rho <\rho_{c}\left(\alpha_{s},\alpha_{t}\right)\\ 1 & \mathrm{if}\;\rho >\rho_{c}\left(\alpha_{s},\alpha_{t}\right), \end{array}\right. \end{array}$$

*where $\rho_{c}\left(\alpha_{s},\alpha_{t}\right)$ is defined in *(11)*.*

The result of Proposition 1, for which the proof can be found in Section 7.4, shows that $\rho_{c}\left(\alpha_{s},\alpha_{t}\right)$ is the phase transition boundary separating the negative transfer regime from the positive transfer regime. When the similarity metric is $\rho <\rho_{c}\left(\alpha_{s},\alpha_{t}\right)$, the optimal transfer ratio is $\delta^{★}=0$, indicating that we should not transfer any source knowledge. Transfer becomes helpful only when ρ moves past the threshold. Note that, for this particular model, there is also an interesting feature that the optimal $\delta^{★}$ jumps to 1 in the positive transfer phase, meaning that we should fully copy the source weight vector.

### 4.3. Sufficient Condition

Next, we consider a binary classification task, where the nonlinear functions φ(·) and $\hat{\varphi }\left(\cdot \right)$ are both the sign function. In this part, we provide a *sufficient* condition for when the hard transfer is beneficial. Before stating our predictions, we need a few definitions related to the Moreau envelope function defined in (15). For simplicity of notation, we refer to the Moreau envelope function as $M_{\ell }\left(\cdot ,\cdot \right)$. Based on [25], $M_{\ell }\left(\cdot ,\cdot \right)$ is differentiable in $R\times R^{+}$. We refer to its derivatives with respect to the first and second arguments as $M_{\ell ,1}^{\prime }\left(\cdot ,\cdot \right)$ and $M_{\ell ,2}^{\prime }\left(\cdot ,\cdot \right)$, respectively. If $M_{\ell }\left(\cdot ,\cdot \right)$ is twice differentiable, we refer to its second derivative with respect to the first and second arguments as $M_{\ell ,1}^{\prime \prime }\left(\cdot ,\cdot \right)$ and $M_{\ell ,2}^{\prime \prime }\left(\cdot ,\cdot \right)$, respectively. Additionally, we refer to its second derivative with respect to the first then the second arguments as $M_{\ell ,12}^{\prime \prime }\left(\cdot ,\cdot \right)$.

We define $q_{0}$, $r_{0}$, and $\sigma_{0}$ as the optimal solutions of the standard learning formulation (i.e., δ=0 in (19)). Moreover, we define the constants $\beta_{1}$ and $\beta_{2}$ as follows:

(21) $$\begin{array}{c} \beta_{1}=\left(1-\rho^{2}\right){\left(q_{s}^{★}\right)}^{2}+{\left(r_{s}^{★}\right)}^{2},\;\beta_{2}=\rho q_{s}^{★}, \end{array}$$

where $q_{s}^{★}$ and $r_{s}^{★}$ are optimal solutions of the deterministic source formulation given in (14). Define the constants $I_{11}$, $I_{12}$, $I_{13}$, and $I_{14}$ as follows:

$$\begin{array}{c} \left\{\begin{array}{c} I_{11}=\alpha_{t}E\left(SHM_{\ell ,1}^{\prime \prime }\left[r_{0}H+q_{0}S;\frac{1}{\sigma_{0}}\right]\right),\;I_{12}=\alpha_{t}E\left(S^{2}M_{\ell ,1}^{\prime \prime }\left[r_{0}H+q_{0}S;\frac{1}{\sigma_{0}}\right]\right)+\lambda \\ I_{13}=-\frac{\alpha_{t}}{\sigma_{0}^{2}}E\left(SM_{\ell ,12}^{\prime \prime }\left[r_{0}H+q_{0}S;\frac{1}{\sigma_{0}}\right]\right);\;I_{14}=-\frac{\alpha_{t}}{\sigma_{0}}E\left(SM_{\ell ,12}^{\prime \prime }\left[r_{0}H+q_{0}S;\frac{1}{\sigma_{0}}\right]\right)+\sigma_{0}\left(q_{0}-\beta_{2}\right), \end{array}\right. \end{array}$$

where Y=φ(S), and *H* and *S* are two independent standard Gaussian random variables. Now, define the constants $I_{21}$, $I_{22}$, $I_{23}$, and $I_{24}$ as follows:

$$\begin{array}{c} \left\{\begin{array}{c} I_{21}=\alpha_{t}E\left(H^{2}M_{\ell ,1}^{\prime \prime }\left[r_{0}H+q_{0}S;\frac{1}{\sigma_{0}}\right]\right)-\sigma_{0}+\lambda ,\;I_{22}=\alpha_{t}E\left(HSM_{\ell ,1}^{\prime \prime }\left[r_{0}H+q_{0}S;\frac{1}{\sigma_{0}}\right]\right)\\ I_{23}=-\frac{\alpha_{t}}{\sigma_{0}^{2}}E\left(HM_{\ell ,12}^{\prime \prime }\left[r_{0}H+q_{0}S;\frac{1}{\sigma_{0}}\right]\right)-r_{0},\;I_{24}=-\frac{\alpha_{t}}{\sigma_{0}}E\left(HM_{\ell ,12}^{\prime \prime }\left[r_{0}H+q_{0}S;\frac{1}{\sigma_{0}}\right]\right). \end{array}\right. \end{array}$$

Finally, define the constants $I_{31}$, $I_{32}$, $I_{33}$, and $I_{34}$ as follows:

$$\begin{array}{c} \left\{\begin{array}{c} I_{31}=-\frac{\alpha_{t}}{\sigma_{0}^{2}}E\left(HM_{\ell ,21}^{\prime \prime }\left[r_{0}H+q_{0}S;\frac{1}{\sigma_{0}}\right]\right)-r_{0},\;I_{32}=-\frac{\alpha_{t}}{\sigma_{0}^{2}}E\left(SM_{\ell ,12}^{\prime \prime }\left[r_{0}H+q_{0}S;\frac{1}{\sigma_{0}}\right]\right)\\ I_{33}=\frac{2\alpha_{t}}{\sigma_{0}^{3}}E\left(M_{\ell ,2}^{\prime }\left[r_{0}H+q_{0}S;\frac{1}{\sigma_{0}}\right]\right)+\frac{\alpha_{t}}{\sigma_{0}^{4}}E\left(M_{\ell ,2}^{\prime \prime }\left[r_{0}H+q_{0}S;\frac{1}{\sigma_{0}}\right]\right)\\ I_{34}=\frac{\alpha_{t}}{\sigma_{0}^{2}}E\left(M_{\ell ,2}^{\prime }\left[r_{0}H+q_{0}S;\frac{1}{\sigma_{0}}\right]\right)+\frac{\alpha_{t}}{\sigma_{0}^{3}}E\left(M_{\ell ,2}^{\prime \prime }\left[r_{0}H+q_{0}S;\frac{1}{\sigma_{0}}\right]\right)+\frac{1}{2}{\left(q_{0}-\beta_{2}\right)}^{2}+\frac{\beta_{1}}{2}. \end{array}\right. \end{array}$$

Now, we are ready to state our sufficient condition.

**Proposition** **2** (Classification)**.** *Assume that the Moreau envelope function is twice continuously differentiable almost everywhere in $R\times R^{+}$ and that the above expectations are all well-defined. Moreover, assume that both φ(·) and $\hat{\varphi }\left(\cdot \right)$ are the sign function. Then,*

(22) $$\begin{array}{c} \delta^{★}>0\;\mathrm{if}\;q_{0}^{\prime }r_{0}-q_{0}r_{0}^{\prime }>0, \end{array}$$

*where $q_{0}^{\prime }$ and $r_{0}^{\prime }$ are solutions to the following linear system of equations:*

(23) $$\begin{array}{c} \left\{\begin{array}{c} I_{11}r_{0}^{\prime }+I_{12}q_{0}^{\prime }+I_{13}\sigma_{0}^{\prime }+I_{14}=0\\ I_{21}r_{0}^{\prime }+I_{22}q_{0}^{\prime }+I_{23}\sigma_{0}^{\prime }+I_{24}=0\\ I_{31}r_{0}^{\prime }+I_{32}q_{0}^{\prime }+I_{33}\sigma_{0}^{\prime }+I_{34}=0. \end{array}\right. \end{array}$$

We prove this result at the end of Section 7. Note that Proposition 2 is valid for a general family of loss functions and general regularization strength λ≥0. For instance, we can see that the results stated in Proposition 2 are valid for the squared loss and the least absolute deviation (LAD) loss, i.e.,

(24) $$\begin{array}{c} \left\{\begin{array}{c} \ell \left(y;x\right)=\frac{1}{2}{\left(1-yx\right)}^{2}\\ \ell (y;x)=|1-yx|. \end{array}\right. \end{array}$$

Unlike (20), the result in (22) only provides a *sufficient* condition for when the hard transfer is beneficial. Nevertheless, our numerical simulations show that the sufficient condition in (22) provides a good prediction of the phase transition boundary for the majority of parameter settings.

## 5. Remarks

### 5.1. Learning Formulations

Given that the target task predicts the new label with $\hat{\varphi }\left(\cdot \right)$, it is more natural to consider loss functions satisfying the following form:

$$\ell (y_{i};\hat{\varphi }\left(a_{i}^{⊤}w\right)).$$

In this case, the convexity assumption is not necessarily satisfied since the loss function can be viewed as the composition of a convex function with a nonlinear function. To guarantee the convexity, we need additional assumptions on the function $\hat{\varphi }\left(\cdot \right)$. Moreover, note that, once the convexity is guaranteed, the function $\hat{\varphi }\left(\cdot \right)$ can be absorbed by the loss function ℓ(·;·).

### 5.2. Transition from Negative to Positive Transfer

Our first simulation example in Figure 2 shows that the optimal transfer rate $\delta^{★}$ can be 1 while the similarity ρ is still less than 1. Here, we provide an intuitive explanation of this behavior.

Given that the source and target feature vectors are generated from the same distribution, one can see that the source labels can be equivalently expressed as follows:

(25) $$\begin{array}{c} y_{s,i}=\varphi \left(\rho a_{t,i}^{⊤}\xi_{t}+z_{i}\right),\;\forall i\in \left\{1,\ldots ,n_{s}\right\}, \end{array}$$

where ${\left\{z_{i}\right\}}_{i=1}^{n_{s}}$ is an additive noise caused by the mismatch between the source and target hidden vectors. Moreover, note that the noise strength depends on the similarity measure ρ.

First, consider the case when the number of source samples is bigger than the number of target samples (i.e., $\alpha_{s}>\alpha_{t}$). We can see that a large value of ρ means that the source and target models are very closely related. Then, one can expect that the additional available data in the source task will be capable of defeating the effects of noise in (25) for large values of ρ. Specifically, it is expected in this regime that the source model will perform better than the standard learning formulation for values of ρ close to 1. However, as we decrease the similarity ρ, the source model will have a small information about the target data. Then, the performance of the hard formulation is expected to be lower than the standard formulation for small values of ρ. In this regime, the source information may hurt the generalization performance of the target task. Then, we need to only transfer a portion of the source information (see Figure 2a). In some settings, the transition is sharp, which means that the source information is irrelevant for the target task when ρ is smaller than a threshold (see Figure 2b).

Second, consider the case when the number of source samples is smaller than the number of target samples (i.e., $\alpha_{s}<\alpha_{t}$). Given the observation in (25), the performance of the standard method is expected to be better than the hard formulation for all possible values of ρ in this regime (see Figure 7).

## 6. Additional Simulation Results

In this section, we provide additional simulation examples to confirm our asymptotic analysis and illustrate the phase transition phenomenon. In our experiments, we focus on the regression and classification models.

### 6.1. Model Assumptions

For the regression model, we assume that the source, target, and test data are generated according to

(26) $$\begin{array}{c} y_{i}=\max\left(a_{i}^{⊤}\xi ,0\right),\;\forall i\in \left\{1,\ldots ,n\right\}. \end{array}$$

The data ${\left\{\left(a_{i},y_{i}\right)\right\}}_{i=1}^{n}$ can be the training data of the source or target tasks. In this regression model, we assume that the function $\hat{\varphi }\left(.\right)$ is the identity function, i.e., $\hat{\varphi }\left(x\right)=x$. Then, the generalization error corresponding to the soft formulation converges in probability as follows:

$$\begin{array}{c} E_{\mathrm{test}}\overset{p\to \infty }{\to }v-2cq_{t}^{★}+\left({\left(q_{t}^{★}\right)}^{2}+{\left(r_{t}^{★}\right)}^{2}\right), \end{array}$$

where *c* and *v* are defined as follows

$$\begin{array}{cc} \; & c=E\left[z\max\left(z,0\right)\right],\;v=E\left[\max{\left(z,0\right)}^{2}\right], \end{array}$$

where *z* is a standard Gaussian random variable and $q_{t}^{★}$ and $r_{t}^{★}$ are defined in Theorem 1. Additionally, the asymptotic limit of the generalization error corresponding to the hard formulation can be expressed in a similar fashion.

For the binary classification model, we assume that the source, target, and test data labels are binary and generated as follows:

(27) $$\begin{array}{c} y_{i}=\mathrm{sign}\left(a_{i}^{⊤}\xi \right),\;\forall i\in \left\{1,\ldots ,n\right\}, \end{array}$$

where the data ${\left\{\left(a_{i},y_{i}\right)\right\}}_{i=1}^{n}$ can be the training data of the source and target tasks. In this classification model, the objective is to predict the correct sign of any unseen sample $y_{\mathrm{new}}$. Then, we fix the function $\hat{\varphi }\left(.\right)$ to be the sign function. Following Theorem 1, it can be easily shown that the generalization error corresponding to the soft formulation given in (8) converges in probability as follows:

$$\begin{array}{c} E_{\mathrm{test}}\overset{p\to \infty }{\to }\frac{1}{\pi }\cos^{-1}\left(\frac{q_{t}^{★}}{\sqrt{{\left(q_{t}^{★}\right)}^{2}+{\left(r_{t}^{★}\right)}^{2}}}\right). \end{array}$$

Here, $q_{t}^{★}$ and $r_{t}^{★}$ are optimal solutions of the target scalar formulation given in (16). The generalization error corresponding to the hard formulation given in (6) can be expressed in a similar fashion.

### 6.2. Phase Transitions in the Hard Formulation

**Figure 3:**
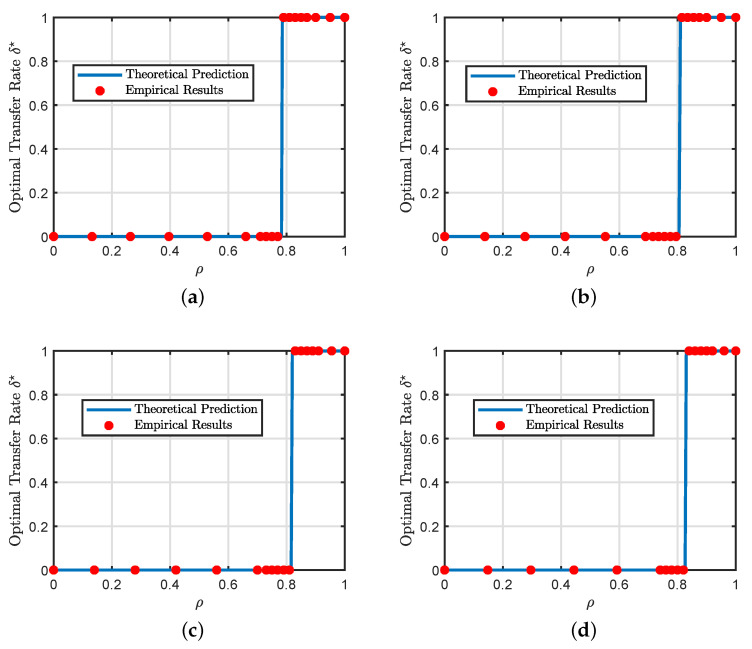
Additional illustrations of the phase transition phenomenon. (**a**) Regression (squared loss, $\alpha_{t}=0.5$, and $\alpha_{s}=3\alpha_{t}$) (**b**) Regression (squared loss, $\alpha_{t}=2$, and $\alpha_{s}=2\alpha_{t}$) (**c**) Binary classification (squared loss, $\alpha_{t}=1.5$, and $\alpha_{s}=3\alpha_{t}$) (**d**) Binary classification (hinge loss, $\alpha_{t}=1.5$, and $\alpha_{s}=3\alpha_{t}$). In all the experiments, we set the regularization strength to be λ=0.1. The blue line represents our theoretical predictions of the optimal transfer rate obtained by solving our asymptotic results in Section 4 for multiple values of δ. The empirical results are averaged over 100 independent Monte Carlo trials with p=2500.

In Section 4, we presented analytical formulas for the phase transition phenomenon but only for the special case of squared loss with no regularization. The main purpose of this experiment, shown in Figure 3, is to demonstrate that the phase transition phenomenon still takes place in more general settings with different loss functions and regularization strengths.

In all the cases shown in Figure 3, the transition from negative to positive transfer is a discontinuous jump from standard learning (i.e., no transfer) to full source transfer. Additionally, Figure 3c,d show that the loss function has a small effect on the phase transition boundary.

### 6.3. Sufficient Condition for the Hard Formulation

**Figure 4:**
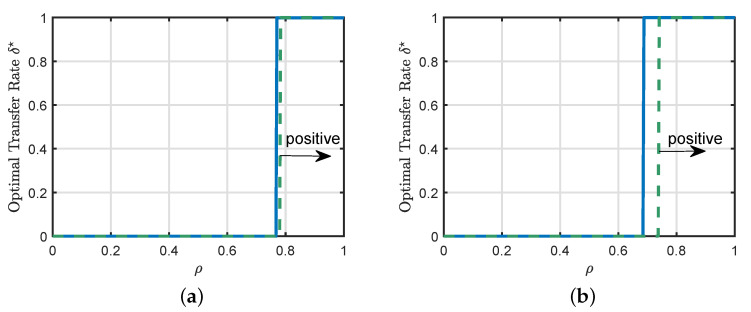
Illustrations of the sufficient condition in Proposition 2. (**a**) Classification (squared loss, $\alpha_{t}=1.5$, and $\alpha_{s}=8\alpha_{t}$) (**b**) Classification (LAD loss, $\alpha_{t}=1.5$, and $\alpha_{s}=8\alpha_{t}$). In all the experiments, we set the regularization strength to be λ=0.1. The blue line represents our theoretical predictions of the optimal transfer rate obtained by solving our asymptotic results in Section 4 for multiple values of δ. The green line represents our sufficient condition for positive transfer stated in Proposition 2.

In Section 4, we presented a sufficient condition for positive transfer. This sufficient condition is valid for a general family of loss functions and a general regularization strength. The main purpose of this experiment, shown in Figure 4, is to illustrate the precision of the sufficient condition for two particular loss functions, i.e., the squared loss and LAD loss.

In all the cases shown in Figure 4, we can see that the transition from negative to positive transfer is a discontinuous jump from standard learning to full source transfer. Additionally, Figure 4a,b show that the sufficient condition summarized in Proposition 2 provides a good prediction of the phase transition boundary for the considered setting.

### 6.4. Soft Transfer: Impact of the Weighting Matrix and Regularization Strength

In this experiment, we empirically explore the impact of the weighting matrix Σ on the generalization error corresponding to the soft formulation. We focus on the binary classification problem with logistic loss. The weighting matrix in (8) takes the following form:

(28) $$\Sigma =\sqrt{\beta_{t}}V,$$

where V is a diagonal matrix generated in three different ways. (1) *Soft Identity*: V is an identity matrix; (2) *Soft Uniform*: the diagonal entries of V are drawn independently from the uniform distribution and then scaled to have their mean equal to 1; and (3): *Soft Beta*: similar to (2), but with the diagonal entries drawn from the beta distribution, followed by rescaling to the unit mean.

**Figure 5:**
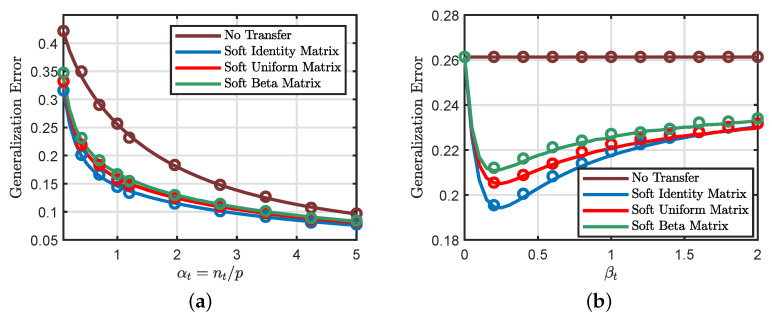
Continuous line: theoretical predictions. Circles: numerical simulations. (**a**) $\alpha_{s}=6\alpha_{t}$, λ=0.1, $\beta_{t}=1/10$, and ρ=0.9. (**b**) $\alpha_{t}=1$, $\alpha_{s}=5\alpha_{t}$, λ=0.3, and ρ=0.75. In all the experiments, we consider the binary classification problem with the logistic loss function. The empirical results are averaged over 50 independent Monte Carlo trials, and we set p=1000.

Figure 5a shows that the considered weighting matrix choices have similar generalization performances, with the identity matrix being slightly better than the other alternatives. Moreover, Figure 5b illustrates the effects of the parameter $\beta_{t}$ in (28) on the generalization performance. It points to the interesting possibility of “designing” the optimal weight matrix to minimize the generalization error.

### 6.5. Soft and Hard Transfer Comparison

In this simulation example, we consider the regression model and compare the performances of the hard and soft transfer formulations as functions of $\alpha_{t}$ and ρ.

**Figure 6:**
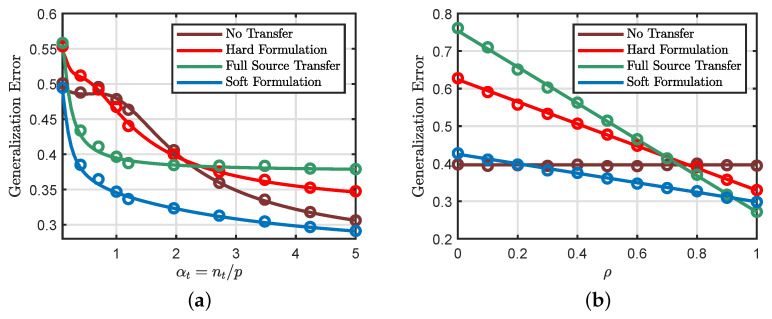
Continuous line: theoretical predictions. Circles: numerical simulations. (**a**) $\alpha_{s}=12\alpha_{t}$, λ=0.2, and ρ=0.75. (**b**) $\alpha_{t}=1.5$, $\alpha_{s}=8\alpha_{t}$, and λ=0.4. In all the experiments, we consider the regression setting with a squared loss. The hard transfer formulation uses δ=0.5, and the soft transfer formulation uses an identity weighting matrix. The empirical results are averaged over 50 independent Monte Carlo trials and we set p=1000.

Figure 6a shows that the soft formulation provides the best generalization performance for all values of $\alpha_{t}$. Moreover, we can see that the hard transfer formulation is only useful for small values $\alpha_{t}$. Figure 6b shows that the performance of the soft and hard transfer formulations depend on the similarity between the source and target tasks. Specifically, the generalization performances of different transfer approaches all improve as we increase the similarity measure ρ. We can also see that the full source transfer approach provides the lowest generalization error when the similarity measure is close to 1, while the soft transfer method leads to the best generalization performance at moderate values of the similarity measure. At very small values of ρ, which means that the two tasks share little resemblance, the standard learning method (i.e., no transfer) is the best scheme one should use.

### 6.6. Effects of the Source Parameters

In the last simulation example, we consider the regression and classification models. We study the performance of the hard and soft transfer formulations when $\alpha_{s}<\alpha_{t}$.

**Figure 7:**
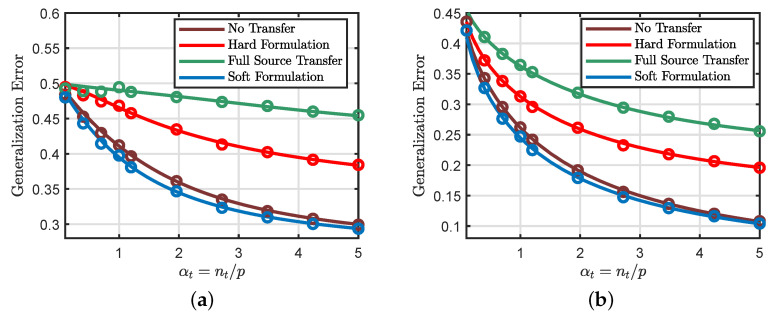
Continuous line: theoretical predictions. Circles: numerical simulations. (**a**) $\alpha_{s}=0.5\alpha_{t}$, λ=0.6, and ρ=0.7. We consider the regression setting with a squared loss. (**b**) $\alpha_{s}=0.5\alpha_{t}$, λ=0.3, and ρ=0.8. We consider the classification setting with a logistic loss. The hard transfer formulation uses δ=0.5, and the soft transfer formulation uses an identity weighting matrix. The empirical results are averaged over 60 independent Monte Carlo trials, and we set p=1000.

Figure 7a considers the regression model. It first shows that the soft transfer formulation provides a slightly better generalization performance compared to the standard method. This behavior can be explained by the fact that the soft formulation requires the target weight vector to be close and not necessarily equal to the source weight vector. Additionally, the source model carries some information about the target task.

We can also see that the hard transfer approach is not beneficial when the number of source samples is smaller than the number of target samples. This result can be explained by the fact that the hard formulation restricts some entries in the target weight vector to be exactly equal to the corresponding entries in the source weight vector. Moreover, the source model is not perfectly aligned with the target model and has smaller data than the target model (see Section 5.2).

The same behavior can be observed in Figure 7b, which considers the classification model.

## 7. Technical Details

In this section, we provide a detailed proof of Theorem 1, and Proportions 1 and 2. Specifically, we focus on analyzing the generalized formulation in (8) using the CGMT framework introduced in the following part.

### 7.1. Technical Tool: Convex Gaussian Min–Max Theorem

The CGMT provides an asymptotic equivalent formulation of primary optimization (PO) problems of the following form:

(29) $$\Phi_{p}\left(G\right)=\min_{w\in S_{w}}\max_{u\in S_{u}}u^{⊤}Gw+\psi \left(w,u\right).$$

Specifically, the CGMT shows that the PO given in (29) is asymptotically equivalent to the following formulation:

(30) $$\;\phi_{p}\left(g,h\right)=\min_{w\in S_{w}}\max_{u\in S_{u}}∥u∥g^{⊤}w+∥w∥h^{⊤}u+\psi \left(w,u\right),$$

referred to as the auxiliary optimization (AO) problem. Before showing the equivalence between PO and AO, the CGMT assumes that $G\in R^{n\times p}$, $g\in R^{p}$, and $h\in R^{n}$; that all have independent and identically distributed standard normal entries; that the feasibility sets $S_{w}\subset R^{p}$ and $S_{u}\subset R^{n}$ are convex and compact; and that the function $\psi \left(.,.\right):R^{p}\times R^{n}\to R$ is continuous *convex-concave* on $S_{w}\times S_{u}$. Moreover, the function ψ(.,.) is independent of the matrix G. Under these assumptions, the CGMT [17] (Theorem 6.1) shows that, for any χ∈R and ζ>0, the following holds:

(31) $$P\left(|\Phi_{p}\left(G\right)-\chi |>\zeta \right)\leq 2P\left(|\phi_{p}\left(g,h\right)-\chi |>\zeta \right).$$

Additionally, the CGMT [17] (Theorem 6.1) provides the following conditions under which the optimal solutions of the PO and AO concentrates around the same set.

**Theorem** **2** (CGMT Framework)**.** *Consider an open set $S_{p}$. Moreover, define the set $S_{p}^{c}=S_{w}\backslash S_{p}$. Let $\phi_{p}$ and $\phi_{p}^{c}$ be the optimal cost values of AO formulation in *(30)* with feasibility sets $S_{w}$ and $S_{p}^{c}$, respectively. Assume that the following properties are all satisfied:*

*(1)*  *There exists a constant ϕ such that the optimal cost $\phi_{p}$ converges in probability to ϕ as p goes to +∞.*
*(2)*  *There exists a positive constant ζ>0 such that $\phi_{p}^{c}\geq \phi +\zeta$ with probability going to 1 as p→+∞.*

*Then, the following convergence in probability holds:*

$$|\Phi_{p}-\phi_{p}|\overset{p\to +\infty }{⟶}0,\;\mathrm{and}\;P\left({\hat{w}}_{p}\in S_{p}\right)\overset{p\to +\infty }{⟶}1,,$$

*where $\Phi_{p}$ and ${\hat{w}}_{p}$ are the optimal cost and the optimal solution of the PO formulation in *(29)*.*

Theorem 2 allows us to analyze the generally easy AO problem to infer the asymptotic properties of the generally hard PO problem. Next, we use the CGMT to rigorously prove the technical results presented in Theorem 1.

### 7.2. Precise Analysis of the Source Formulation

The source formulation defined in (4) is well-studied in recent literature [26]. Specifically, it has been rigorously proven that the performance of the source formulation can be fully characterized after solving the following scalar formulation:

(32) $$\begin{array}{cc} \min_{\begin{array}{c} q_{s},r_{s}\geq 0 \end{array}}\underset{\begin{array}{c} \sigma_{s}>0 \end{array}}{\sup} & \;\alpha_{s}E\left[M_{\ell (Y_{s},.)}\left(r_{s}H_{s}+q_{s}S_{s};\frac{r_{s}}{\sigma_{s}}\right)\right]\\ \; & -\frac{r_{s}\sigma_{s}}{2}+\frac{\lambda }{2}\left(q_{s}^{2}+r_{s}^{2}\right), \end{array}$$

where $Y_{s}=\varphi \left(S_{s}\right)$, and $H_{s}$ and $S_{s}$ are two independent standard Gaussian random variables. The expectation in (32) is taken over the random variables $H_{s}$ and $S_{s}$. Furthermore, the function $M_{\ell (Y_{s},.)}$ introduced in the scalar optimization problem (32) is the Moreau envelope function defined in (15).

### 7.3. Precise Analysis of the Soft Transfer Approach

In this part, we provide a precise asymptotic analysis of the generalized transfer formulation given in (8). Specifically, we focus on analyzing the following formulation:

(33) $$\begin{array}{cc} \min_{w\in R^{p}} & \frac{1}{p}\sum_{i=1}^{n_{t}}\ell \left(y_{i};a_{i}^{⊤}w\right)+\frac{\lambda }{2}{∥w∥}^{2}+\frac{1}{2}{∥\Sigma \left(w-{\hat{w}}_{s}\right)∥}^{2}, \end{array}$$

where ${\hat{w}}_{s}$ is the optimal solution of the source formulation given in (4). Note that the vector ${\hat{w}}_{s}$ is independent of the training data of the target task. For simplicity of notation, we denote by ${\left\{\left(a_{i},y_{i}\right)\right\}}_{i=1}^{n_{t}}$ the training data of the target task. Here, we use the CGMT framework introduced in Section 7.1 to precisely analyze the above formulation.

#### 7.3.1. Formulating the Auxiliary Optimization Problem

Our first objective is to rewrite the generalized formulation in the form of the PO problem given in (29). To this end, we introduce additional optimization variables. Specifically, the generalized formulation can be equivalently formulated as follows:

(34) $$\begin{array}{cc} \min_{w\in R^{p}}\max_{u\in R^{n_{t}}} & \;\frac{1}{p}u^{⊤}Aw-\frac{1}{p}\sum_{i=1}^{n_{t}}\ell^{★}\left(y_{i};u_{i}\right)+\frac{\lambda }{2}{∥w∥}^{2}\\ \; & +\frac{1}{2}{∥\Sigma \left(w-{\hat{w}}_{s}\right)∥}^{2}, \end{array}$$

where the optimization vector $u\in R^{n_{t}}$ is formed as $u={\left[u_{1},\ldots ,u_{n_{t}}\right]}^{⊤}$ and the data matrix $A\in R^{n_{t}\times p}$ is given by $A={\left[a_{1},\ldots ,a_{n_{t}}\right]}^{⊤}$. Additionally, the function $\ell^{★}\left(y;.\right)$ denotes the convex conjugate function of the loss function ℓ(y;.). First, observe that the CGMT framework assumes that the feasibility sets of the minimization and maximization problems are compact. Then, our next step is to show that the formulation given in (34) satisfies this assumption.

**Lemma** **1** (Primal-Dual Compactness)**.** *Assume that $\hat{w}$ and $\hat{u}$ are optimal solutions of the optimization problem in *(34)*. Then, there exist two constants $C_{w}>0$ and $C_{u}>0$ such that the following convergence in probability holds:*

(35) $$\begin{array}{c} P\left(∥\hat{w}∥\leq C_{w}\right)\overset{\;p\to +\infty \;}{\to }1,\;P\left(∥\hat{u}∥/\sqrt{n_{t}}\leq C_{u}\right)\overset{\;p\to +\infty \;}{\to }1. \end{array}$$

A detailed proof of Lemma 1 is provided in Appendix B. The proof of the above result follows using Assumption 3 to prove the compactness of the optimal solution $\hat{w}$. Moreover, it uses the asymptotic results in [27] (Theorem 2.1), which provides the concentration properties of the minimum and maximum eigenvalues of random matrices. To show the compactness of the optimal dual vector $\hat{u}$, we use Assumption 3 and the result in [25] (Proposition 11.3), which provides the inversion rules for subgradient relations.

The theoretical result in Lemma 1 shows that the optimization problem in (34) can be equivalently formulated with compact feasibility sets on events with probability going to one. Then, it suffices to study the constrained version of (34). Note that the data labels ${\left\{y_{i}\right\}}_{i=1}^{n_{t}}$ depend on the data matrix A. Then, one can decompose the matrix A as follows:

$$\begin{array}{cc} A & =AP_{\xi_{t}}+AP_{\xi }^{⊥}=A\xi_{t}\xi_{t}^{⊤}+AP_{\xi }^{⊥}, \end{array}$$

where the matrix $P_{\xi_{t}}\in R^{p\times p}$ denotes the projection matrix onto the space spanned by the vector $\xi_{t}$ and the matrix $P_{\xi }^{⊥}=I_{p}-\xi_{t}\xi_{t}^{⊤}$ denotes the projection matrix onto the orthogonal complement of the space spanned by the vector $\xi_{t}$. Note that we can express A as follows without changing its statistics:

(36) $$\begin{array}{c} A=s_{t}\xi_{t}^{⊤}+GP_{\xi }^{⊥}, \end{array}$$

where $s_{t}∼N\left(0,I_{n_{t}}\right)$ and the components of the matrix $G\in R^{n_{t}\times p}$ are drawn independently from a standard Gaussian distribution and where $s_{t}$ and G are independent. Here, (36) represents an equality in distribution. This means that the formulation in (34) can be expressed as follows:

(37) $$\begin{array}{cc} \; & \;\min_{∥w∥\leq C_{w}}\max_{u\in C_{t}}\frac{1}{p}u^{⊤}GP_{\xi }^{⊥}w+\frac{1}{p}u^{⊤}s_{t}\xi_{t}^{⊤}w+\frac{\lambda }{2}{∥w∥}^{2}\\ \; & -\frac{1}{p}\sum_{i=1}^{n_{t}}\ell^{★}\left(y_{i};u_{i}\right)+\frac{1}{2}{∥\Sigma \left(w-{\hat{w}}_{s}\right)∥}^{2}, \end{array}$$

where the set $C_{t}$ is defined as $C_{t}=\left\{u:∥u∥/\sqrt{n_{t}}\leq C_{u}\right\}$. Note that the formulation in (37) is in the form of the primary formulation given in (29). Here, the function ψ(.,.) is defined as follows:

(38) $$\begin{array}{cc} \psi (w,u) & =\frac{1}{p}u^{⊤}s_{t}\xi_{t}^{⊤}w+\frac{\lambda }{2}{∥w∥}^{2}-\frac{1}{p}\sum_{i=1}^{n_{t}}\ell^{★}\left(y_{i};u_{i}\right)\\ \; & +\frac{1}{2}{∥\Sigma \left(w-{\hat{w}}_{s}\right)∥}^{2}. \end{array}$$

One can easily see that the optimization problem in (37) has compact convex feasibility sets. Moreover, the function ψ(.,.) is continuous, convex–concave, and independent of the Gaussian matrix G. This shows that the assumptions of the CGMT are all satisfied by the primary formulation in (37). Then, following the CGMT framework, the auxiliary formulation corresponding to our primary problem in (37) can be expressed as follows:

(39) $$\begin{array}{cc} \; & \;\min_{∥w∥\leq C_{w}}\max_{u\in C_{t}}\frac{∥u∥}{p}g^{⊤}P_{\xi }^{⊥}w+\frac{1}{p}u^{⊤}s_{t}\xi_{t}^{⊤}w+\frac{h^{⊤}u}{p}∥P_{\xi }^{⊥}w∥\\ \; & \;+\frac{\lambda }{2}{∥w∥}^{2}-\frac{1}{p}\sum_{i=1}^{n_{t}}\ell^{★}\left(y_{i};u_{i}\right)+\frac{1}{2}{∥\Sigma \left(w-{\hat{w}}_{s}\right)∥}^{2}, \end{array}$$

where $g\in R^{p}$ and $h\in R^{n_{t}}$ are two independent standard Gaussian vectors. The rest of the proof focuses on simplifying the obtained AO formulation and on studying its asymptotic properties.

#### 7.3.2. Simplifying the AO Problem of the Target Task

Here, we focus on simplifying the auxiliary formulation corresponding to the target task. We start our analysis by decomposing the target optimization vector $w\in R^{p}$ as follows:

(40) $$\begin{array}{c} w=\left(\xi_{t}^{⊤}w\right)\xi_{t}+B_{\xi_{t}}^{⊥}r_{t}, \end{array}$$

where $r_{t}\in R^{p-1}$ is a free vector and $B_{\xi_{t}}^{⊥}\in R^{p\times (p-1)}$ is formed by an orthonormal basis orthogonal to the vector $\xi_{t}$. Now, define the variable $q_{t}$ as follows: $q_{t}=\xi_{t}^{⊤}w$. Based on the result in Lemma 1 and the decomposition in (40), there exist $C_{q_{t}}>0$, $C_{r}>0$, and $C_{u}>0$ such that our auxiliary formulation can be asymptotically expressed in terms of the variables $q_{t}$ and $r_{t}$ as follows:

$$\begin{array}{cc} \; & \min_{\begin{array}{c} \left(q_{t},r_{t}\right)\in T_{1} \end{array}}\max_{\begin{array}{c} u\in C_{t} \end{array}}\frac{∥u∥}{p}g^{⊤}B_{\xi_{t}}^{⊥}r_{t}+\frac{∥r_{t}∥}{p}h^{⊤}u+\frac{q_{t}}{p}u^{⊤}s_{t}+\frac{\lambda }{2}q_{t}^{2}\\ \; & +\frac{\lambda }{2}{∥r_{t}∥}^{2}-\frac{1}{p}\sum_{i=1}^{n_{t}}\ell^{★}\left(y_{i};u_{i}\right)+\frac{1}{2}q_{t}^{2}V_{p,t}-q_{t}V_{p,ts}\\ \; & +\frac{1}{2}r_{t}^{⊤}{\left(B_{\xi_{t}}^{⊥}\right)}^{⊤}\Lambda B_{\xi_{t}}^{⊥}r_{t}+q_{t}\xi_{t}^{⊤}\Lambda B_{\xi_{t}}^{⊥}r_{t}-r_{t}^{⊤}{\left(B_{\xi_{t}}^{⊥}\right)}^{⊤}\Lambda {\hat{w}}_{s}. \end{array}$$

Here, we drop terms independent of the optimization variables and the matrix $\Lambda \in R^{p\times p}$ is defined as $\Lambda =\Sigma^{⊤}\Sigma$. Additionally, the feasibility set $T_{1}$ is defined as follows:

(41) $$\begin{array}{c} T_{1}=\left\{\left(q_{t},r_{t}\right):\left|q_{t}\right|\leq C_{q_{t}},∥r_{t}∥\leq C_{r}\right\}. \end{array}$$

Here, the sequence of random variables $V_{p,t}$ and $V_{p,ts}$ are defined as follows:

(42) $$\begin{array}{cc} \; & V_{p,t}=\xi_{t}^{⊤}\Lambda \xi_{t},\;V_{p,ts}=\xi_{t}^{⊤}\Lambda {\hat{w}}_{s}. \end{array}$$

Next, we focus on simplifying the obtained auxiliary formulation. Our strategy is to solve over the direction of the optimization vector $r\in R^{p-1}$. This step requires an interchange between non-convex minimization and non-concave maximization. We can justify the interchange using the theoretical result in [17] (Lemma A.3). The main argument in [17] (Lemma A.3) is that the strong convexity of the primary formulation in (37) allows us to perform such an interchange in the corresponding auxiliary formulation. The optimization problem over the vector $r_{t}$ with fixed norm, i.e., $∥r_{t}∥=r_{t}$, can be formulated as follows:

(43) $$\begin{array}{cc} C_{p}^{★}= & \min_{r_{t}\in R^{p-1}}\;b_{p}^{⊤}r_{t}+\frac{1}{2}r_{t}^{⊤}\Lambda^{⊥}r_{t},\;\;s.t.\;\;∥r_{t}∥=r_{t}. \end{array}$$

Here, we ignore constant terms independent of $r_{t}$, and the matrix $\Lambda^{⊥}\in R^{\left(p-1)\times (p-1\right)}$ and the vector $b_{p}\in R^{p-1}$ can be expressed as follows:

$$\begin{array}{cc} \Lambda^{⊥}={\left(B_{\xi_{t}}^{⊥}\right)}^{⊤}\Lambda B_{\xi_{t}}^{⊥},\;b_{p} & =\frac{∥u∥}{p}{\left(B_{\xi_{t}}^{⊥}\right)}^{⊤}g+q_{t}{\left(B_{\xi_{t}}^{⊥}\right)}^{⊤}\Lambda \xi_{t}^{⊤}\\ \; & -{\left(B_{\xi_{t}}^{⊥}\right)}^{⊤}\Lambda {\hat{w}}_{s}. \end{array}$$

The optimization problem in (43) is non-convex given the norm equality constraint. It is well-studied in the literature [28] and is known as the trust region subproblem. Using the same analysis as in [20], the optimal cost value of the optimization problem (43) can be expressed in terms of a one-dimensional optimization problem as follows:

(44) $$\begin{array}{c} \;C_{p}^{★}=\underset{\sigma_{t}>-\mu_{p}}{\sup}\left\{-\frac{1}{2}b_{p}^{⊤}{\left[\Lambda^{⊥}+\sigma_{t}I_{p-1}\right]}^{-1}b_{p}-\frac{\sigma_{t}r_{t}^{2}}{2}\right\}, \end{array}$$

where $\mu_{p}$ is the minimum eigenvalue of the matrix $\Lambda^{⊥}$, denoted by $\sigma_{\min}\left(\Lambda^{⊥}\right)$. This result can be seen by equivalently formulating the non-convex problem in (43) as follows:

$$\begin{array}{cc} C_{p}^{★}= & \min_{r_{t}\in R^{p-1}}\max_{\sigma_{t}\in R}\;b_{p}^{⊤}r_{t}+\frac{1}{2}r_{t}^{⊤}\Lambda^{⊥}r_{t}+\frac{\sigma_{t}}{2}\left({∥r_{t}∥}^{2}-r_{t}^{2}\right). \end{array}$$

Then, we show that the optimal $\sigma_{t}$ satisfies a constraint that preserves the convexity over $r_{t}$. This allows us to interchange the maximization and minimization and to solve over the vector $r_{t}$. The above analysis shows that the AO formulation corresponding to our primary problem can be expressed as follows:

(45) $$\begin{array}{cc} \; & \min_{\begin{array}{c} \left(q_{t},r_{t}\right)\in T_{2} \end{array}}\max_{\begin{array}{c} u\in C_{t} \end{array}}\underset{\sigma_{t}>-\mu_{p}}{\sup}\frac{r_{t}}{p}h^{⊤}u+\frac{q_{t}}{p}u^{⊤}s_{t}+\frac{\lambda }{2}q_{t}^{2}+\frac{\lambda }{2}r_{t}^{2}\\ \; & -\frac{1}{p}\sum_{i=1}^{n_{t}}\ell^{★}\left(y_{i};u_{i}\right)+\frac{1}{2}q_{t}^{2}V_{p,t}-q_{t}V_{p,ts}-\frac{{∥u∥}^{2}}{2p}T_{p,g}\left(\sigma_{t}\right)\\ \; & -\frac{\sigma_{t}r_{t}^{2}}{2}-\frac{1}{2}q_{t}^{2}T_{p,t}\left(\sigma_{t}\right)-\frac{1}{2}T_{p,s}\left(\sigma_{t}\right)+q_{t}T_{p,ts}\left(\sigma_{t}\right), \end{array}$$

where the set $T_{2}$ has the same definition as the set $T_{1}$ except that we replace $∥r_{t}∥$ with $r_{t}$. Here, the sequence of random functions $T_{p,g}\left(.\right)$, $T_{p,t}\left(.\right)$, $T_{p,s}\left(.\right)$, and $T_{p,ts}\left(.\right)$ can be expressed as follows:

$$\begin{array}{c} \left\{\begin{array}{c} T_{p,g}\left(\sigma_{t}\right)=\frac{1}{p}g^{⊤}B_{\xi_{t}}^{⊥}{\left[\Lambda^{⊥}+\sigma_{t}I_{p-1}\right]}^{-1}{\left(B_{\xi_{t}}^{⊥}\right)}^{⊤}g\\ T_{p,t}\left(\sigma_{t}\right)=\xi_{t}^{⊤}\Lambda B_{\xi_{t}}^{⊥}{\left[\Lambda^{⊥}+\sigma_{t}I_{p-1}\right]}^{-1}{\left(B_{\xi_{t}}^{⊥}\right)}^{⊤}\Lambda \xi_{t}\\ T_{p,s}\left(\sigma_{t}\right)={\hat{w}}_{s}^{⊤}\Lambda B_{\xi_{t}}^{⊥}{\left[\Lambda^{⊥}+\sigma_{t}I_{p-1}\right]}^{-1}{\left(B_{\xi_{t}}^{⊥}\right)}^{⊤}\Lambda {\hat{w}}_{s}\\ T_{p,ts}\left(\sigma_{t}\right)=\xi_{t}^{⊤}\Lambda B_{\xi_{t}}^{⊥}{\left[\Lambda^{⊥}+\sigma_{t}I_{p-1}\right]}^{-1}{\left(B_{\xi_{t}}^{⊥}\right)}^{⊤}\Lambda {\hat{w}}_{s}. \end{array}\right. \end{array}$$

Note that the formulation in (45) is obtained after dropping terms that converge in probability to zero. This simplification can be justified using a similar analysis to that in [20] (Lemma 3). The main idea in [20] (Lemma 3) is to show that both loss functions converge uniformly to the same limit.

Next, the objective is to simplify the obtained AO formulation over the optimization vector $u\in R^{n_{t}}$. Based on the property stated in [20] (Lemma 4), the optimization over the vector u can be expressed as follows:

$$\begin{array}{cc} I_{p}^{★} & =\max_{\begin{array}{c} u\in C_{t} \end{array}}r_{t}h^{⊤}u+q_{t}u^{⊤}s_{t}-\sum_{i=1}^{n_{t}}\ell^{★}\left(y_{i};u_{i}\right)-\frac{{∥u∥}^{2}}{2}T_{p,g}\left(\sigma_{t}\right)\\ \; & =\sum_{i=1}^{n_{t}}M_{\ell (y_{i},.)}\left(r_{t}h_{i}+q_{t}s_{t,i};T_{p,g}\left(\sigma_{t}\right)\right). \end{array}$$

This result is valid on events with probability going to one as *p* goes to +∞. Here, the function $M_{\ell (y_{i},.)}$ is the Moreau envelope function defined in (15). The proof of this property is omitted since it follows the same ideas as [20] (Lemma 4). The main idea in [20] (Lemma 4) is to use Assumption 3 to show that the optimal solution of the unconstrained version of the maximization problem is bounded asymptotically and then to use the property introduced in [25] (Example 11.26) to complete the proof. Now, our auxiliary formulation can be asymptotically simplified to a scalar optimization problem as follows:

(46) $$\begin{array}{cc} \; & \min_{\begin{array}{c} \left(q_{t},r_{t}\right)\in T_{2} \end{array}}\underset{\sigma_{t}>-\mu_{p}}{\sup}\frac{\lambda }{2}\left(q_{t}^{2}+r_{t}^{2}\right)-\frac{\sigma_{t}r_{t}^{2}}{2}-\frac{1}{2}q_{t}^{2}Z_{p,t}\left(\sigma_{t}\right)-\frac{1}{2}Z_{p,s}\left(\sigma_{t}\right)\\ \; & +\frac{1}{p}\sum_{i=1}^{n_{t}}M_{\ell (y_{i},.)}\left(r_{t}h_{i}+q_{t}s_{t,i};T_{p,g}\left(\sigma_{t}\right)\right)+q_{t}Z_{p,ts}\left(\sigma_{t}\right), \end{array}$$

where the functions $Z_{p,t}\left(\cdot \right)$, $Z_{p,ts}\left(\cdot \right)$, and $Z_{p,s}\left(\cdot \right)$ are defined as follows:

(47) $$\begin{array}{cc} \; & Z_{p,t}\left(\sigma_{t}\right)=T_{p,t}\left(\sigma_{t}\right)-V_{p,t},\;Z_{p,ts}\left(\sigma_{t}\right)=T_{p,ts}\left(\sigma_{t}\right)-V_{p,ts} \end{array}$$

(48) $$\begin{array}{cc} \; & Z_{p,s}\left(\sigma_{t}\right)=T_{p,s}\left(\sigma_{t}\right)-V_{p,s},\;\mathrm{where}\;V_{p,s}={\hat{w}}_{s}^{⊤}\Lambda {\hat{w}}_{s}. \end{array}$$

Note that the auxiliary formulation in (46) now has scalar optimization variables. Then, it remains to study its asymptotic properties. We refer to this problem as the target scalar formulation.

#### 7.3.3. Asymptotic Analysis of the Target Scalar Formulation

In this part, we study the asymptotic properties of the target scalar formulation expressed in (46). We start our analysis by studying the asymptotic properties of the sequence of random functions $T_{p,g}\left(.\right)$, $Z_{p,t}\left(.\right)$, $Z_{p,s}\left(.\right)$, and $Z_{p,ts}\left(.\right)$ as given in the following lemma.

**Lemma** **2** (Asymptotic Properties)**.** *First, the random variable $\mu_{p}$ converges in probability to $\mu_{\min}$, where $\mu_{\min}$ is defined in Assumption 5. For any fixed σ>0, the following convergence in probability holds true:*

$$\begin{array}{c} \left\{\begin{array}{c} Z_{p,t}\left(\sigma -\mu_{p}\right)\overset{p\to +\infty }{\to }Z_{t}\left(\sigma -\mu_{\min}\right)\\ Z_{p,ts}\left(\sigma -\mu_{p}\right)\overset{p\to +\infty }{\to }Z_{ts}\left(\sigma -\mu_{\min}\right)\\ Z_{p,s}\left(\sigma -\mu_{p}\right)\overset{p\to +\infty }{\to }Z_{s}\left(\sigma -\mu_{\min}\right)\\ T_{p,g}\left(\sigma -\mu_{p}\right)\overset{p\to +\infty }{\to }T_{g}\left(\sigma -\mu_{\min}\right)=T_{1}\left(\sigma -\mu_{\min}\right). \end{array}\right. \end{array}$$

*Here, the deterministic functions $Z_{t}\left(.\right)$, $Z_{ts}\left(.\right)$, $Z_{s}\left(.\right)$, $T_{1}\left(.\right)$, and $T_{3}\left(.\right)$ are defined as follows:*

$$\begin{array}{c} \left\{\begin{array}{c} Z_{t}\left(\sigma \right)=\sigma -1/T_{1}\left(\sigma \right),\;Z_{ts}\left(\sigma \right)=\rho q_{s}^{★}Z_{t}\left(\sigma \right)\\ Z_{s}\left(\sigma \right)=\left(\left(1-\rho^{2}\right){\left(q_{s}^{★}\right)}^{2}+{\left(r_{s}^{★}\right)}^{2}\right)T_{3}\left(\sigma \right)+{\left(\rho q_{s}^{★}\right)}^{2}Z_{t}\left(\sigma \right)\\ T_{1}\left(\sigma \right)=E_{\mu }\left[1/\left(\mu +\sigma \right)\right],\;T_{3}\left(\sigma \right)=-E_{\mu }\left[\mu \sigma /(\mu +\sigma )\right]. \end{array}\right. \end{array}$$

*Moreover, the constants $q_{s}^{★}$ and $r_{s}^{★}$ are optimal solutions of the source asymptotic formulation defined in *(32)*.*

A detailed proof of Lemma 2 is provided in Appendix C. Now that we obtained the asymptotic properties of the sequence of random variables, it remains to study the asymptotic properties of the optimal cost and optimal solution set of the scalar formulation in (46). To state our first asymptotic result, we define the following deterministic optimization problem:

(49) $$\begin{array}{cc} \; & \min_{\begin{array}{c} \left(q_{t},r_{t}\right)\in T_{2} \end{array}}\underset{\sigma_{t}>-\mu_{\min}}{\sup}\;\frac{\lambda }{2}\left(q_{t}^{2}+r_{t}^{2}\right)-\frac{\sigma_{t}r_{t}^{2}}{2}-\frac{1}{2}Z_{s}\left(\sigma_{t}\right)-\frac{1}{2}q_{t}^{2}Z_{t}\left(\sigma_{t}\right)\\ \; & +\alpha_{t}E\left[M_{\ell (Y_{t},.)}\left(r_{t}H_{t}+q_{t}S_{t};T_{g}\left(\sigma_{t}\right)\right)\right]+q_{t}Z_{ts}\left(\sigma_{t}\right), \end{array}$$

where $H_{t}$ and $S_{t}$ are two independent standard Gaussian random variables and $Y_{t}=\varphi \left(S_{t}\right)$. Here, the function $M_{\ell (Y_{t},.)}$ denotes the Moreau envelope function defined in (15) and the expectation is taken over the random variables $H_{t}$ and $S_{t}$, and the possibly random function φ(.). Now, we are ready to state our asymptotic property of the cost function of (46).

**Lemma** **3** (Cost Function of the Traget AO Formulation)**.** *Define $O_{p,t}\left(.\right)$ as the loss function of the target scalar optimization problem given in *(46)*. Additionally, define $O_{t}\left(.\right)$ as the cost function of the deterministic formulation in *(49)*. Then, the following convergence in probability holds true:*

(50) $$\begin{array}{c} O_{p,t}\left(q_{t},r_{t},\sigma_{t}-\mu_{p}\right)\overset{\;\;p\to +\infty \;\;}{\to }O_{t}\left(q_{t},r_{t},\sigma_{t}-\mu_{\min}\right), \end{array}$$

*for any fixed feasible $q_{t}$, $r_{t}$, and $\sigma_{t}>0$.*

The proof of the asymptotic property stated in Lemma 3 uses the asymptotic results stated in Lemma 2. Moreover, it uses the weak law of large numbers to show that the empirical mean of the Moreau envelope concentrates around its expected value. Based on Assumption 3, one can see that the following pointwise convergence is valid:

$$\begin{array}{c} \frac{1}{n_{t}}\sum_{i=1}^{n_{t}}M_{\ell (y_{i},.)}\left(r_{t}h_{i}+q_{t}s_{t,i};x\right)\overset{p\to +\infty }{\to }E\left[M_{\ell (Y,.)}\left(r_{t}H+q_{t}S;x\right)\right], \end{array}$$

where *H* and *S* are independent standard Gaussian random variables and Y=φ(S). The above property is valid for any x>0, $r_{t}\geq 0$, and $q_{t}$. Based on [25] (Theorem 2.26), the Moreau envelope function is convex and continuously differentiable with respect to x>0. Combining this with [29] (Theorem 7.46), the above asymptotic function is continuous in x>0. Then, using Lemma 2, the uniform convergence, and the continuity property, we conclude that the empirical average of the Moreau envelope converges in probability to the following function:

(51) $$\begin{array}{c} E\left[M_{\ell (Y,.)}\left(r_{t}H+q_{t}S;T_{g}\left(\sigma_{t}-\mu_{\min}\right)\right)\right], \end{array}$$

for any fixed feasible $q_{t}$, $r_{t}$, and $\sigma_{t}>0$. This completes the proof of Lemma 3.

Before continuing our analysis, we provide the convexity properties of the cost function of the deterministic problem in (49) in the following lemma.

**Lemma** **4** (Strong Conexity)**.** *Define $O_{t}\left(\cdot ,\cdot ,\cdot \right)$ as the cost function of the optimization problem in *(49)*. Then, $O_{t}\left(\cdot ,\cdot ,\cdot \right)$ is concave in the maximization variable $\sigma_{t}$ for any fixed feasible $\left(q_{t},r_{t}\right)$. Moreover, define the function $O_{t}\left(\cdot ,\cdot \right)$ as follows:*

(52) $$\begin{array}{c} O_{t}\left(q_{t},r_{t}\right)=\underset{\sigma_{t}>-\mu_{\min}}{\sup}f\left(q_{t},r_{t},\sigma_{t}\right). \end{array}$$

*Then, the function $O_{t}\left(\cdot ,\cdot \right)$ is strongly convex in the minimization variables $\left(q_{t},r_{t}\right)$.*

The proof of Lemma 4 is provided in Appendix D. Now, we use these properties to show that the optimal solution set of the formulation in (46) converges in probability to the optimal solution set of the formulation in (49).

**Lemma** **5** (Consistency of the Target AO Formulation)**.** *Define $P_{p,t}$ and $P_{t}$ as the optimal set of $\left(q_{t},r_{t}\right)$ of the optimization problems formulated in *(46)* and *(49)*. Moreover, define $O_{p,t}^{★}$ and $O_{t}^{★}$ as the optimal cost values of the optimization problems formulated in *(46)* and *(49)*. Then, the following converges in probability holds true:*

(53) $$\begin{array}{c} O_{p,t}^{★}\overset{p\to +\infty }{\to }O_{t}^{★},\;D\left(P_{p,t},P_{t}\right)\overset{p\to +\infty }{\to }0, \end{array}$$

*where D(A,B) denotes the deviation between the sets A and B and is defined as $D\left(A,B\right)=\sup_{c_{1}\in A}\inf_{c_{2}\in B}∥c_{1}-c_{2}∥$.*

The stated result can be proven by first observing that the loss function $O_{t}\left(.\right)$ corresponding to the deterministic formulation in (49) satisfies the following:

(54) $$\begin{array}{c} \lim_{\sigma_{t}\to +\infty }O_{t}\left(q_{t},r_{t},\sigma_{t}-\mu_{\min}\right)=-\infty \end{array}$$

for any $r_{t}>0$ and any fixed $q_{t}$. Combining this with the convergence result in Lemma 3, ref. [17] (Lemma B.1), and [17] (Lemma B.2), we obtain the following asymptotic result:

$$\begin{array}{c} \underset{\sigma_{t}>0}{\sup}O_{p,t}\left(q_{t},r_{t},\sigma_{t}-\mu_{p}\right)\overset{\;\;p\to +\infty \;\;}{\to }\underset{\sigma_{t}>0}{\sup}O_{t}\left(q_{t},r_{t},\sigma_{t}-\mu_{\min}\right). \end{array}$$

Here, the results in [17] (Lemma B.1) and [17] (Lemma B.2) provide convergence properties of minimization problems over open sets. Note that, if $r_{t}=0$, the supremum in the above convergence result occurs at $\sigma_{t}\to +\infty$. However, it can be checked that the above convergence result still holds. Based on Lemma 4, the cost function of the minimization problem in (49) is strongly convex in $\left(q_{t},r_{t}\right)$. Moreover, the feasibility set of the minimization problem is convex and compact. Additionally, the cost function of the minimization problem in (49) is continuous in the feasibility set. Then, using the results in [30] (Theorem II.1) and [31] (Theorem 2.1), we obtain the convergence properties stated in Lemma 5. Here, the results in [30] (Theorem II.1) and [31] (Theorem 2.1) provide uniform convergence and consistency properties of convex optimization problems.

Now that we obtained the asymptotic problem, it remains to study the asymptotic properties of the training and generalization errors corresponding to the target formulation in (8).

#### 7.3.4. Specialization to Hard Formulation

Before starting the analysis of the generalization error, we specialize our general analysis to the hard transfer formulation. First, note that δ=1 implies that the hard transfer formulation is equivalent to the source formulation. Next, we assume that δ<1. To obtain the asymptotic limit of the hard formulation, we specialize the general results in (49) to the following probability distribution:

(55) $$\begin{array}{c} P_{p}\left(\mu \right)=\left\{\begin{array}{cc} 0 & \mathrm{with}\;\mathrm{probability}\;(1-\delta )\\ +\infty & \mathrm{with}\;\mathrm{probability}\;\delta . \end{array}\right. \end{array}$$

Note that the probability distribution in (55) satisfies Assumption 5. Then, the asymptotic limit of the soft formulation corresponding to the probability distribution $P_{\mu }\left(.\right)$, defined in (55), can be expressed as follows:

(56) $$\begin{array}{cc} \min_{\begin{array}{c} \left(q_{t},r_{t}\right)\in T_{2} \end{array}}\underset{\sigma_{t}>0}{\sup} & \;\frac{\lambda }{2}\left(q_{t}^{2}+r_{t}^{2}\right)+\frac{\sigma_{t}\delta }{2}\left(\left(1-\rho^{2}\right){\left(q_{s}^{★}\right)}^{2}+{\left(r_{s}^{★}\right)}^{2}\right)\\ \; & \;+\alpha_{t}E\left[M_{\ell (Y_{t},.)}\left(r_{t}H_{t}+q_{t}S_{t};\frac{1-\delta }{\sigma_{t}}\right)\right]-\frac{\sigma_{t}r_{t}^{2}}{2}\\ \; & \;+\frac{\sigma_{t}\delta }{2(1-\delta )}{\left(q_{t}-\rho q_{s}^{★}\right)}^{2}. \end{array}$$

This shows that the asymptotic limit of the hard formulation is the deterministic problem (56).

#### 7.3.5. Asymptotic Analysis of the Training and Generalization Errors

First, the generalization error corresponding to the target task is given by

(57) $$\begin{array}{cc} E_{\mathrm{test}} & =\frac{1}{4^{\upsilon }}E\left[{\left(\varphi \left(a_{t,\mathrm{new}}^{⊤}\xi_{t}\right)-\hat{\varphi }\left({\hat{w}}_{t}^{⊤}a_{t,\mathrm{new}}\right)\right)}^{2}\right], \end{array}$$

where $a_{t,\mathrm{new}}$ is an unseen target feature vector. Now, consider the following two random variables

$$\begin{array}{c} \nu_{1}=a_{t,\mathrm{new}}^{⊤}\xi_{t},\;\mathrm{and}\;\nu_{2}={\hat{w}}_{t}^{⊤}a_{t,\mathrm{new}}. \end{array}$$

Given ${\hat{w}}_{t}$ and $\xi_{t}$, the random variables $\nu_{1}$ and $\nu_{2}$ have a bivaraite Gaussian distribution with zero mean vector and covariance matrix given as follows:

(58) $$\begin{array}{c} C_{p}=\left[\begin{array}{cc} {∥\xi_{t}∥}^{2} & \xi_{t}^{⊤}{\hat{w}}_{t}\\ \xi_{t}^{⊤}{\hat{w}}_{t} & {∥{\hat{w}}_{t}∥}^{2} \end{array}\right]. \end{array}$$

To precisely analyze the asymptotic behavior of the generalization error, it suffices to analyze the properties of the covariance matrix $C_{p}$. Define the random variables ${\hat{q}}_{p,t}^{★}$ and ${\hat{r}}_{p,t}^{★}$ for the target task as follows:

(59) $$\begin{array}{cc} \; & {\hat{q}}_{p,t}^{★}=\xi_{t}^{⊤}{\hat{w}}_{t},\;\mathrm{and}\;{\hat{r}}_{p,t}^{★}=∥{\left(B_{\xi_{t}}^{⊥}\right)}^{⊤}{\hat{w}}_{t}∥, \end{array}$$

where $B_{\xi_{t}}^{⊥}$ is defined in Section 7.3.2. Then, the covariance matrix $C_{p}$ given in (58) can be expressed as follows:

$$\begin{array}{c} \left[\begin{array}{cc} 1 & {\hat{q}}_{p,t}^{★}\\ {\hat{q}}_{p,t}^{★} & {\left({\hat{q}}_{p,t}^{★}\right)}^{2}+{\left({\hat{r}}_{p,t}^{★}\right)}^{2} \end{array}\right]. \end{array}$$

Hence, to study the asymptotic properties of the generalization error, it suffices to study the asymptotic properties of the random quantities ${\hat{q}}_{p,t}^{★}$ and ${\hat{r}}_{p,t}^{★}$.

**Lemma** **6** (Consistency of the Target Formulation)**.** *The random quantities ${\hat{q}}_{p,t}^{★}$ and ${\hat{r}}_{p,t}^{★}$ satisfy the following asymptotic properties:*

$$\begin{array}{c} {\hat{q}}_{p,t}^{★}\overset{p\to +\infty }{\to }q_{t}^{★},\;\mathrm{and}\;{\hat{r}}_{p,t}^{★}\overset{p\to +\infty }{\to }r_{t}^{★}, \end{array}$$

*where $q_{t}^{★}$ and $r_{t}^{★}$ are the optimal solutions of the deterministic formulation stated in *(49)*.*

To prove the above asymptotic result, we define ${\tilde{q}}_{p,t}^{★}$ and ${\tilde{r}}_{p,t}^{★}$ as follows:

(60) $$\begin{array}{cc} \; & {\tilde{q}}_{p,t}^{★}=\xi_{t}^{⊤}{\tilde{w}}_{t},\;\mathrm{and}\;{\tilde{r}}_{p,t}^{★}=∥{\left(B_{\xi_{t}}^{⊥}\right)}^{⊤}{\tilde{w}}_{t}∥, \end{array}$$

where ${\tilde{w}}_{t}$ is the optimal solution of the auxiliary formulation in (39). Given the result in Lemma 5 and the analysis in Section 7.3.2 and Section 7.3.3, the convergence result in Lemma 5 is also satisfied by our auxiliary formulation in (39), i.e.,

$$\begin{array}{c} {\tilde{q}}_{p,t}^{★}\overset{p\to +\infty }{\to }q_{t}^{★},\;\mathrm{and}\;{\tilde{r}}_{p,t}^{★}\overset{p\to +\infty }{\to }r_{t}^{★}. \end{array}$$

The rest of the proof of the convergence result stated in Lemma 6 is based on the CGMT framework, i.e., Theorem 2. Specifically, it follows after showing that the assumptions in Theorem 2 are all satisfied. First, we define the set $S_{p}$ in Theorem 2 as follows:

(61) $$\begin{array}{c} S_{p}=\{w\in R^{p}:|\xi_{t}^{⊤}w-q_{t}^{★}|<\epsilon \}\cup \{w\in R^{p}:|∥{\left(B_{\xi_{t}}^{⊥}\right)}^{⊤}w∥-r_{t}^{★}\left|<\epsilon \right\}, \end{array}$$

where $q_{t}^{★}$ and $r_{t}^{★}$ are the optimal solutions of the deterministic formulation stated in (49). Note that the cost function of the problem (49) is strongly convex in the minimization variables. Based on the analysis in the previous sections, note that the feasibility sets of the problems defined in Theorem 2 are compact asymptotically. Moreover, the analysis in the previous sections shows that there exists a constant ϕ such that the optimal cost $\phi_{p}$ defined in Theorem 2 converges in probability to ϕ as *p* goes to +∞. Additionally, the same analysis in the previous sections shows that there exists a constant $\phi^{c}$ such that the optimal cost $\phi_{p}^{c}$ defined in Theorem 2 converges in probability to $\phi^{c}$ as *p* goes to +∞. The strong convexity property of the cost function of the optimization problem in (49) can then be used to show that there exists ζ>0 such that $\phi^{c}>\phi +\zeta$. This implies that the second assumption in Theorem 2 is satisfied for the considered set $S_{p}$ and any fixed ϵ>0. This then shows that the convergence results in Lemma 6 are all satisfied.

Note that the CGMT framework applied to prove Lemma 6 also shows that the optimal cost value of the soft target formulation in (8) converges in probability to the optimal cost value of the deterministic formulation given in (49). Combining this with the result in Lemma 6 shows the convergence property of the training error stated in (17). Now, it remains to show the convergence of the generalization error. It suffices to show that the generalization error defined in (57) is continuous in the quantities ${\hat{q}}_{p,t}^{★}$ and ${\hat{r}}_{p,t}^{★}$. This follows based on Assumption 4 and the continuity under integral sign property [32]. This shows the convergence result in (18), which completes the proof of Theorem 1. Note that the above analysis of the soft target formulation in (8) is valid for any choice of $C_{q_{t}}$ and $C_{r}$ that satisfy the result in Lemma 1. One can ignore these bounds given the convexity properties of the deterministic formulation in (49). This leads to the scalar formulations introduced in (16) and (19).

### 7.4. Phase Transitions in Hard Formulation

In this part, we provide a rigorous proof of Proposition 1. Here, we consider the squared loss function. In this case, the deterministic source formulation given in (14) can be simplified as follows:

(62) $$\begin{array}{cc} \min_{\begin{array}{c} q_{s},r_{s}\geq 0 \end{array}}\; & \frac{1}{2}\max{\left\{-r_{s}+\sqrt{\alpha_{s}}{\left(q_{s}^{2}+r_{s}^{2}+v_{s}-2q_{s}c_{s}\right)}^{\frac{1}{2}},0\right\}}^{2}\\ \; & +\frac{\lambda }{2}\left(q_{s}^{2}+r_{s}^{2}\right), \end{array}$$

where the constants $v_{s}$ and $c_{s}$ are defined as $v_{s}=E\left[Y_{s}^{2}\right]$ and $c_{s}=E\left[S_{s}Y_{s}\right]$, $Y_{s}=\varphi \left(S_{s}\right)$, and $S_{s}$ is a standard Gaussian random variable. Additionally, the target scalar formulation given in (16) can be simplified as follows:

(63) $$\begin{array}{cc} \min_{\begin{array}{c} q_{t},r_{t}\geq 0 \end{array}}\underset{\sigma_{t}>0}{\sup} & \frac{\lambda }{2}\left(q_{t}^{2}+r_{t}^{2}\right)+\frac{\sigma_{t}\delta }{2}\left(\left(1-\rho^{2}\right){\left(q_{s}^{★}\right)}^{2}+{\left(r_{s}^{★}\right)}^{2}\right)\\ \; & \;+\frac{\alpha_{t}\sigma_{t}}{2\left(1-\delta \right)+2\sigma_{t}}\left(r_{t}^{2}+q_{t}^{2}+v_{t}-2q_{t}c_{t}\right)-\frac{\sigma_{t}r_{t}^{2}}{2}\\ \; & \;+\frac{\sigma_{t}\delta }{2(1-\delta )}{\left(q_{t}-\rho q_{s}^{★}\right)}^{2}, \end{array}$$

where the constants $v_{t}$ and $c_{t}$ are defined as $v_{t}=E\left[Y_{t}^{2}\right]$ and $c_{t}=E\left[Y_{t}S_{t}\right]$, $Y_{t}=\varphi \left(S_{t}\right)$, and $S_{t}$ is a standard Gaussian random variable. Under the conditions stated in Proposition 1, the source deterministic formulation given in (62) can be simplified as follows:

(64) $$\begin{array}{cc} \min_{\begin{array}{c} q_{s},r_{s}\geq 0 \end{array}}\; & -r_{s}+\sqrt{\alpha_{s}}{\left(q_{s}^{2}+r_{s}^{2}+v_{s}-2q_{s}c_{s}\right)}^{\frac{1}{2}}. \end{array}$$

Note that one can easily solve the variables $q_{s}$ and $r_{s}$. Specifically, the optimal solutions of (64) can be expressed as follows:

(65) $$\begin{array}{c} q_{s}^{★}=c_{s},\;\mathrm{and}\;r_{s}^{★}=\sqrt{v_{s}-c_{s}^{2}}/\sqrt{\alpha_{s}-1}. \end{array}$$

Moreover, the target deterministic formulation given in (63) can be expressed as follows:

(66) $$\begin{array}{cc} \min_{\begin{array}{c} q_{t},r_{t}\geq 0 \end{array}}\underset{\sigma_{t}>0}{\sup} & \frac{\sigma_{t}\delta }{2}\beta_{2}+\frac{\alpha_{t}\sigma_{t}}{2\left(1-\delta \right)+2\sigma_{t}}\left(r_{t}^{2}+q_{t}^{2}+v_{t}-2q_{t}c_{t}\right)-\frac{\sigma_{t}r_{t}^{2}}{2}\\ \; & +\frac{\sigma_{t}\delta }{2(1-\delta )}{\left(q_{t}-\beta_{1}\right)}^{2}, \end{array}$$

where $\beta_{1}$ and $\beta_{2}$ are given by

(67) $$\begin{array}{c} \beta_{1}=\rho q_{s}^{★},\;\beta_{2}=\left(\left(1-\rho^{2}\right){\left(q_{s}^{★}\right)}^{2}+{\left(r_{s}^{★}\right)}^{2}\right). \end{array}$$

Before solving the optimization problem in (66), we consider the following change in variable:

(68) $$\begin{array}{c} x_{t}^{2}+r_{t}^{2}-\delta \beta_{2}-\frac{\delta }{1-\delta }{\left(q_{t}-\beta_{1}\right)}^{2}. \end{array}$$

Note that the above change in variable is valid since the formulation in (66) requires the left-hand side of (68) to be positive. Therefore, the formulation in (66) can be expressed in terms of $x_{t}$ instead of $r_{t}$ as follows:

(69) $$\begin{array}{cc} \min_{\begin{array}{c} q_{t},x_{t}\geq 0 \end{array}}\underset{\sigma_{t}>0}{\sup} & \frac{\alpha_{t}\sigma_{t}}{2\left(1-\delta \right)+2\sigma_{t}}\left(x_{t}^{2}+\delta \beta_{2}+\frac{\delta }{1-\delta }{\left(q_{t}-\beta_{1}\right)}^{2}+q_{t}^{2}+v_{t}-2q_{t}c_{t}\right)-\frac{\sigma_{t}x_{t}^{2}}{2}. \end{array}$$

Now, it can be easily checked that the above optimization problem can be solved over the variable $\sigma_{t}$ to give the following formulation:

$$\begin{array}{cc} \min_{\begin{array}{c} q_{t},x_{t}\geq 0 \end{array}} & \frac{1}{2}\max{\left\{-x_{t}\sqrt{1-\delta }+\sqrt{\alpha_{t}}{\left(x_{t}^{2}+\delta \beta_{2}+\frac{\delta }{1-\delta }{\left(q_{t}-\beta_{1}\right)}^{2}+q_{t}^{2}+v_{t}-2q_{t}c_{t}\right)}^{\frac{1}{2}},0\right\}}^{2}. \end{array}$$

It is now clear that one can solve the problem in (69) in closed form. Moreover, it can be easily checked that the optimal solutions of the optimization problem (66) can be expressed as follows:

$$\begin{array}{c} \left\{\begin{array}{c} q_{t}^{★}=\left(1-\delta \right)c_{t}+\delta \beta_{1}\\ {\left(r_{t}^{★}\right)}^{2}=\frac{1-\delta }{\alpha_{t}+\delta -1}\left(\left(\delta -1\right)c_{t}^{2}+\delta \beta_{1}^{2}+\delta \beta_{2}+v_{t}-2\delta \beta_{1}c_{t}\right)+\delta \beta_{2}+\delta \left(1-\delta \right){\left(c_{t}-\beta_{1}\right)}^{2}. \end{array}\right. \end{array}$$

Then, the asymptotic limit of the generalization error corresponding to the hard formulation can be determined in closed-form. Given that the source and target models given in (1) and (2) use the same data-generating function, the constants $v_{t}$, $c_{t}$, $v_{s}$, and $c_{s}$ are all equal. We express them as *v* and *c* in the rest of the proof.

Next, we assume that the function $\hat{\varphi }\left(.\right)$ is the identity function. Based on the asymptotic result stated in Corollary 1, the asymptotic limit of the generalization error corresponding to the hard formulation can be expressed as follows:

$$\begin{array}{cc} E_{\mathrm{test}} & =v-2cq_{t}^{★}+{\left(q_{t}^{★}\right)}^{2}+{\left(r_{t}^{★}\right)}^{2}. \end{array}$$

It can be easily checked that the generalization error can be express as follows:

(70) $$\begin{array}{cc} E_{\mathrm{test}} & =\frac{\alpha_{t}}{\alpha_{t}+\delta -1}\left(\delta \left\{{\left(c-\beta_{1}\right)}^{2}+\beta_{2}\right\}+\left(v-c^{2}\right)\right). \end{array}$$

Note that the generalization error obtained above depends explicitly on δ. Now, it suffices to study the derivative of $E_{\mathrm{test}}$ to find the properties of the optimal transfer rate δ that minimizes the generalization error. Note that the derivative can be expressed as follows:

(71) $$\begin{array}{c} \;E_{\mathrm{test}}^{\prime }\left(\delta \right)=\frac{\left(\alpha_{t}-1\right)\left\{{\left(c-\beta_{1}\right)}^{2}+\beta_{2}\right\}-\left(v-c^{2}\right)}{{\left(\alpha_{t}+\delta -1\right)}^{2}}. \end{array}$$

This shows that the derivative of the generalization error has the same sign as the numerator. This means that the optimal transfer rate satisfies the following:

(72) $$\begin{array}{c} \delta^{★}=\left\{\begin{array}{cc} 1 & \mathrm{if}\;Z_{t}<0\\ 0 & \mathrm{if}\;Z_{t}>0\\ \left[0\;1\right] & \mathrm{otherwise}, \end{array}\right. \end{array}$$

where $Z_{t}$ is given by

(73) $$\begin{array}{c} Z_{t}=\left(\alpha_{t}-1\right)\left\{{\left(c-\beta_{1}\right)}^{2}+\beta_{2}\right\}-\left(v-c^{2}\right). \end{array}$$

It can be easily shown that the condition in (72) can be expressed as the one given in (20). This completes the proof of Proposition 1.

### 7.5. Sufficient Condition for the Hard Formulation

In this part, we provide a rigorous proof to Proposition 2. Suppose that the assumptions in Proposition 2 are all satisfied. Additionally, we assume that the function $\hat{\varphi }\left(.\right)$ is the sign function. Based on the asymptotic result stated in Corollary 1, the asymptotic limit of the generalization error corresponding to the hard formulation can be expressed as follows:

(74) $$\begin{array}{cc} E_{\mathrm{test}}\left(\delta \right) & =\frac{1}{\pi }\mathrm{acos}\left(\frac{q_{t}\left(\delta \right)}{\sqrt{{\left(q_{t}\left(\delta \right)\right)}^{2}+{\left(r_{t}\left(\delta \right)\right)}^{2}}}\right), \end{array}$$

where $q_{t}\left(\delta \right)$ and $r_{t}\left(\delta \right)$ are optimal solutions to the deterministic problem in (19) for fixed δ. A simple sufficient condition for positive transfer is when $E_{\mathrm{test}}\left(\delta \right)$ is decreasing at δ=0. This means that there exists some δ>0 such that the transfer learning method introduced in (6) is better than the standard method when the following function increases at δ=0:

(75) $$\begin{array}{c} g\left(\delta \right)=\frac{q_{t}\left(\delta \right)}{\sqrt{{\left(q_{t}\left(\delta \right)\right)}^{2}+{\left(r_{t}\left(\delta \right)\right)}^{2}}}. \end{array}$$

After computing the derivative of the function g(·) at zero, one can see that the transfer learning method introduced in (6) is better than the standard method when the following condition is true:

(76) $$\begin{array}{c} q_{t}^{\prime }\left(0\right)r_{t}\left(0\right)-q_{t}\left(0\right)r_{t}^{\prime }\left(0\right)>0, \end{array}$$

where $q_{t}\left(0\right)$ and $r_{t}\left(0\right)$ denote the optimal solutions of the standard learning formulation (i.e., δ=0 in (19)). Additionally, $q_{t}^{\prime }\left(0\right)$ and $r_{t}^{\prime }\left(0\right)$ denote the derivative of the functions $q_{t}\left(\delta \right)$ and $r_{t}\left(\delta \right)$ at δ=0. The above analysis shows that it suffices to find the values of $q_{t}^{\prime }\left(0\right)$ and $r_{t}^{\prime }\left(0\right)$ to fully characterize the sufficient condition in (76). Before stating our analysis, we define $\beta_{1}$ and $\beta_{2}$ as follows:

(77) $$\begin{array}{c} \beta_{1}=\left(\left(1-\rho^{2}\right){\left(q_{s}^{★}\right)}^{2}+{\left(r_{s}^{★}\right)}^{2}\right),\;\beta_{2}=\rho q_{s}^{★}, \end{array}$$

where $q_{s}^{★}$ and $r_{s}^{★}$ are the optimal solutions of the deterministic source formulation given in (14).

Note that the optimal solution of the deterministic formulation in (19) satisfy the following system of equations:

$$\begin{array}{c} \left\{\begin{array}{c} \alpha_{t}E\left(SM_{\ell ,1}^{\prime }\left[r_{t}\left(\delta \right)H+q_{t}\left(\delta \right)S;\frac{1-\delta }{\sigma_{t}\left(\delta \right)}\right]\right)+\frac{\delta \sigma_{t}\left(\delta \right)}{1-\delta }\left(q_{t}\left(\delta \right)-\beta_{2}\right)+\lambda q_{t}\left(\delta \right)=0\\ \alpha_{t}E\left(HM_{\ell ,1}^{\prime }\left[r_{t}\left(\delta \right)H+q_{t}\left(\delta \right)S;\frac{1-\delta }{\sigma_{t}\left(\delta \right)}\right]\right)-\sigma_{t}\left(\delta \right)r_{t}\left(\delta \right)+\lambda r_{t}\left(\delta \right)=0\\ \frac{\delta }{2}\beta_{1}-\frac{\alpha_{t}\left(1-\delta \right)}{\sigma_{t}{\left(\delta \right)}^{2}}E\left(M_{\ell ,2}^{\prime }\left[r_{t}\left(\delta \right)H+q_{t}\left(\delta \right)S;\frac{1-\delta }{\sigma_{t}\left(\delta \right)}\right]\right)-\frac{r_{t}{\left(\delta \right)}^{2}}{2}+\frac{\delta }{2(1-\delta )}{\left(q_{t}\left(\delta \right)-\beta_{2}\right)}^{2}=0. \end{array}\right. \end{array}$$

The derivative of the first equation at δ=0 can be expressed as follows:

(78) $$\begin{array}{cc} \; & \alpha_{t}E\left(\left(SHr^{\prime }\left(0\right)+S^{2}q^{\prime }\left(0\right)\right)M_{\ell ,1}^{\prime \prime }\left[r_{t}\left(0\right)H+q_{t}\left(0\right)S;\frac{1}{\sigma_{t}\left(0\right)}\right]\right)+\sigma_{t}\left(0\right)\left(q_{t}\left(0\right)-\beta_{2}\right)\\ \; & -\frac{\alpha_{t}}{\sigma_{t}{\left(0\right)}^{2}}\left(\sigma_{t}\left(0\right)+\sigma_{t}^{\prime }\left(0\right)\right)E\left(SM_{\ell ,12}^{\prime \prime }\left[r_{t}\left(0\right)H+q_{t}\left(0\right)S;\frac{1}{\sigma_{t}\left(0\right)}\right]\right)+\lambda q_{t}^{\prime }\left(0\right)=0, \end{array}$$

where $q_{t}\left(0\right)$, $r_{t}\left(0\right)$, and $\sigma_{t}\left(0\right)$ denote optimal solutions of the standard learning formulation (i.e., δ=0 in (19)). This means that they are known. Moreover, $q_{t}^{\prime }\left(0\right)$, $r_{t}^{\prime }\left(0\right)$ and $\sigma_{t}^{\prime }\left(0\right)$ are unknown and denote the derivative of the functions $q_{t}\left(\delta \right)$, $r_{t}\left(\delta \right)$, and $\sigma_{t}\left(\delta \right)$ at δ=0. Now, define the constants $I_{11}$, $I_{12}$, $I_{13}$, and $I_{14}$ as follows:

(79) $$\begin{array}{c} \left\{\begin{array}{c} I_{11}=\alpha_{t}E\left(SHM_{\ell ,1}^{\prime \prime }\left[r_{t}\left(0\right)H+q_{t}\left(0\right)S;\frac{1}{\sigma_{t}\left(0\right)}\right]\right)\\ I_{12}=\alpha_{t}E\left(S^{2}M_{\ell ,1}^{\prime \prime }\left[r_{t}\left(0\right)H+q_{t}\left(0\right)S;\frac{1}{\sigma_{t}\left(0\right)}\right]\right)+\lambda \\ I_{13}=-\frac{\alpha_{t}}{\sigma_{t}{\left(0\right)}^{2}}E\left(SM_{\ell ,12}^{\prime \prime }\left[r_{t}\left(0\right)H+q_{t}\left(0\right)S;\frac{1}{\sigma_{t}\left(0\right)}\right]\right)\\ I_{14}=-\frac{\alpha_{t}}{\sigma_{t}\left(0\right)}E\left(SM_{\ell ,12}^{\prime \prime }\left[r_{t}\left(0\right)H+q_{t}\left(0\right)S;\frac{1}{\sigma_{t}\left(0\right)}\right]\right)+\sigma_{t}\left(0\right)\left(q_{t}\left(0\right)-\beta_{2}\right). \end{array}\right. \end{array}$$

This means that the equation in (78) can be expressed as follows:

(80) $$\begin{array}{c} I_{11}r_{t}^{\prime }\left(0\right)+I_{12}q_{t}^{\prime }\left(0\right)+I_{13}\sigma_{t}^{\prime }\left(0\right)+I_{14}=0. \end{array}$$

Similarly, the derivative of the second equation at δ=0 can be expressed as follows:

(81) $$\begin{array}{cc} \; & \alpha_{t}E\left(\left(H^{2}r^{\prime }\left(0\right)+HSq^{\prime }\left(0\right)\right)M_{\ell ,1}^{\prime \prime }\left[r_{t}\left(0\right)H+q_{t}\left(0\right)S;\frac{1}{\sigma_{t}\left(0\right)}\right]\right)-\sigma_{t}^{\prime }\left(0\right)r_{t}\left(0\right)-\sigma_{t}\left(0\right)r_{t}^{\prime }\left(0\right)\\ \; & -\frac{\alpha_{t}}{\sigma_{t}{\left(0\right)}^{2}}\left(\sigma_{t}\left(0\right)+\sigma_{t}^{\prime }\left(0\right)\right)E\left(HM_{\ell ,12}^{\prime \prime }\left[r_{t}\left(0\right)H+q_{t}\left(0\right)S;\frac{1}{\sigma_{t}\left(0\right)}\right]\right)+\lambda r_{t}^{\prime }\left(0\right)=0. \end{array}$$

Now, define the constants $I_{21}$, $I_{22}$, $I_{23}$, and $I_{24}$ as follows:

(82) $$\begin{array}{c} \left\{\begin{array}{c} I_{21}=\alpha_{t}E\left(H^{2}M_{\ell ,1}^{\prime \prime }\left[r_{t}\left(0\right)H+q_{t}\left(0\right)S;\frac{1}{\sigma_{t}\left(0\right)}\right]\right)-\sigma_{t}\left(0\right)+\lambda \\ I_{22}=\alpha_{t}E\left(HSM_{\ell ,1}^{\prime \prime }\left[r_{t}\left(0\right)H+q_{t}\left(0\right)S;\frac{1}{\sigma_{t}\left(0\right)}\right]\right)\\ I_{23}=-\frac{\alpha_{t}}{\sigma_{t}{\left(0\right)}^{2}}E\left(HM_{\ell ,12}^{\prime \prime }\left[r_{t}\left(0\right)H+q_{t}\left(0\right)S;\frac{1}{\sigma_{t}\left(0\right)}\right]\right)-r_{t}\left(0\right)\\ I_{24}=-\frac{\alpha_{t}}{\sigma_{t}\left(0\right)}E\left(HM_{\ell ,12}^{\prime \prime }\left[r_{t}\left(0\right)H+q_{t}\left(0\right)S;\frac{1}{\sigma_{t}\left(0\right)}\right]\right). \end{array}\right. \end{array}$$

This means that the equation in (81) can be expressed as follows:

(83) $$\begin{array}{c} I_{21}r_{t}^{\prime }\left(0\right)+I_{22}q_{t}^{\prime }\left(0\right)+I_{23}\sigma_{t}^{\prime }\left(0\right)+I_{24}=0. \end{array}$$

Moreover, the derivative of the third equation at δ=0 can be expressed as follows:

(84) $$\begin{array}{cc} \; & \frac{\beta_{1}}{2}+\frac{\alpha_{t}}{\sigma_{t}{\left(0\right)}^{3}}\left(\sigma_{t}\left(0\right)+2\sigma_{t}^{\prime }\left(0\right)\right)E\left(M_{\ell ,2}^{\prime }\left[r_{t}\left(0\right)H+q_{t}\left(0\right)S;\frac{1}{\sigma_{t}\left(0\right)}\right]\right)-r_{t}^{\prime }\left(0\right)r_{t}\left(0\right)\\ \; & -\frac{\alpha_{t}}{\sigma_{t}{\left(0\right)}^{2}}E\left(\left(Hr^{\prime }\left(0\right)+Sq^{\prime }\left(0\right)\right)M_{\ell ,21}^{\prime \prime }\left[r_{t}\left(0\right)H+q_{t}\left(0\right)S;\frac{1}{\sigma_{t}\left(0\right)}\right]\right)+\frac{1}{2}{\left(q_{t}\left(0\right)-\beta_{2}\right)}^{2}\\ \; & +\frac{\alpha_{t}}{\sigma_{t}{\left(0\right)}^{4}}\left(\sigma_{t}\left(0\right)+\sigma_{t}^{\prime }\left(0\right)\right)E\left(M_{\ell ,2}^{\prime \prime }\left[r_{t}\left(0\right)H+q_{t}\left(0\right)S;\frac{1}{\sigma_{t}\left(0\right)}\right]\right)=0. \end{array}$$

We define the constants $I_{31}$, $I_{32}$, $I_{33}$, and $I_{34}$ as follows:

$$\begin{array}{c} \left\{\begin{array}{c} I_{31}=-\frac{\alpha_{t}}{\sigma_{t}{\left(0\right)}^{2}}E\left(HM_{\ell ,21}^{\prime \prime }\left[r_{t}\left(0\right)H+q_{t}\left(0\right)S;\frac{1}{\sigma_{t}\left(0\right)}\right]\right)-r_{t}\left(0\right)\\ I_{32}=-\frac{\alpha_{t}}{\sigma_{t}{\left(0\right)}^{2}}E\left(SM_{\ell ,12}^{\prime \prime }\left[r_{t}\left(0\right)H+q_{t}\left(0\right)S;\frac{1}{\sigma_{t}\left(0\right)}\right]\right)\\ I_{33}=\frac{2\alpha_{t}}{\sigma_{t}{\left(0\right)}^{3}}E\left(M_{\ell ,2}^{\prime }\left[r_{t}\left(0\right)H+q_{t}\left(0\right)S;\frac{1}{\sigma_{t}\left(0\right)}\right]\right)+\frac{\alpha_{t}}{\sigma_{t}{\left(0\right)}^{4}}E\left(M_{\ell ,2}^{\prime \prime }\left[r_{t}\left(0\right)H+q_{t}\left(0\right)S;\frac{1}{\sigma_{t}\left(0\right)}\right]\right)\\ I_{34}=\frac{\alpha_{t}}{\sigma_{t}{\left(0\right)}^{2}}E\left(M_{\ell ,2}^{\prime }\left[r_{t}\left(0\right)H+q_{t}\left(0\right)S;\frac{1}{\sigma_{t}\left(0\right)}\right]\right)+\frac{\alpha_{t}}{\sigma_{t}{\left(0\right)}^{3}}E\left(M_{\ell ,2}^{\prime \prime }\left[r_{t}\left(0\right)H+q_{t}\left(0\right)S;\frac{1}{\sigma_{t}\left(0\right)}\right]\right)\\ \;\;\;\;\;+\frac{1}{2}{\left(q_{t}\left(0\right)-\beta_{2}\right)}^{2}+\frac{\beta_{1}}{2}. \end{array}\right. \end{array}$$

Therefore, the equation in (84) can be expressed as follows:

(85) $$\begin{array}{c} I_{31}r_{t}^{\prime }\left(0\right)+I_{32}q_{t}^{\prime }\left(0\right)+I_{33}\sigma_{t}^{\prime }\left(0\right)+I_{34}=0. \end{array}$$

The above analysis shows that the values of $q_{t}^{\prime }\left(0\right)$ and $r_{t}^{\prime }\left(0\right)$ can be determined after solving the following system of linear equations:

(86) $$\begin{array}{c} \left\{\begin{array}{c} I_{11}r_{t}^{\prime }\left(0\right)+I_{12}q_{t}^{\prime }\left(0\right)+I_{13}\sigma_{t}^{\prime }\left(0\right)+I_{14}=0\\ I_{21}r_{t}^{\prime }\left(0\right)+I_{22}q_{t}^{\prime }\left(0\right)+I_{23}\sigma_{t}^{\prime }\left(0\right)+I_{24}=0\\ I_{31}r_{t}^{\prime }\left(0\right)+I_{32}q_{t}^{\prime }\left(0\right)+I_{33}\sigma_{t}^{\prime }\left(0\right)+I_{34}=0, \end{array}\right. \end{array}$$

over the three unknowns $q_{t}^{\prime }\left(0\right)$, $r_{t}^{\prime }\left(0\right)$, and $\sigma_{t}^{\prime }\left(0\right)$. This completes the proof of Proposition 2.

## 8. Conclusions

In this paper, we presented a precise characterization of the asymptotic properties of two simple transfer learning formulations. Specifically, our results show that the training and generalization errors corresponding to the considered transfer formulations converge to deterministic functions. These functions can be explicitly found by combining the solutions of two deterministic scalar optimization problems. Our simulation results validate our theoretical predictions and reveal the existence of a phase transition phenomenon in the hard transfer formulation. Specifically, it shows that the hard transfer formulation moves from negative transfer to positive transfer when the similarity of the source and target tasks move past a well-defined critical threshold.

## Data Availability

Not applicable

## Appendix A. Technical Assumptions

Note that Assumption 1 is essential to show that the soft formulation in (4) concentrates in the large system limit. It also guarantees that the vectors $\xi_{t}\in R^{p}$ and $\xi_{s}\in R^{p}$ have correlations equal to ρ, asymptotically. This is aligned with the definition in (3). Assumption 4 is also introduced to guarantee that the generalization error concentrates in the large system limit. It is satisfied by popular regression and classification models. For instance, observe that the conditions in Assumption 4 are all satisfied by the regression model considering φ:x→max(x,0). Moreover, they are satisfied by the binary classification model considering φ:x→sign(x).

The analysis presented in this paper mostly focuses on regularized transfer learning formulations (i.e., λ>0). The convexity properties in Assumption 3 are essential to apply the CGMT framework. Moreover, the properties in (12) are used to guarantee the compactness assumptions in the CGMT framework (see Theorem 2). In this appendix, we check the validity of Assumption 3 using popular loss functions, i.e., squared loss for regression tasks and logistic and hinge losses for binary classification tasks. To this end, assume that *C* is an arbitrary fixed positive constant.

1. Squared loss: It is easy to see that the squared loss is a proper strongly convex function in R, where 1 is a strong convexity parameter. Moreover, L(·) and its sub-differential set ∂L(·) can be expressed as follows:
  (A1) $$\begin{array}{c} L\left(v\right)=\frac{1}{2}{∥v-y∥}^{2},\;\;\partial L\left(v\right)=\left\{v-y\right\}, \end{array}$$
  where the vector y is formed by the concatenation of ${\left\{y_{i}\right\}}_{i=1}^{n_{t}}$. Then, there exists R>0 such that
  (A2) $$\begin{array}{c} \underset{∥v∥\leq C\sqrt{n_{t}}}{\sup}\left|L(v)\right|\leq Rn_{t},\;\;\underset{∥v∥\leq C\sqrt{n_{t}}}{\sup}\;\underset{s\in \partial L(v)}{\sup}∥s∥=\underset{∥v∥\leq C\sqrt{n_{t}}}{\sup}∥v-y∥\leq R\sqrt{n_{t}}, \end{array}$$
  with probability going to 1 as *p* grow to +∞. The inequality follows using the regularity condition in Assumption 4 and the weak law of large numbers. Then, the squared loss satisfies Assumption 3 for any λ≥0.
2. Logistic loss: Now, we consider the logistic loss applied to a binary classification model (i.e., $y_{i}\in \left\{-1,1\right\}$). Note that the logistic loss is a proper convex function in R. Moreover, L(·) and its sub-differential set ∂L(·) are given by
  (A3) $$L\left(v\right)=\sum_{i=1}^{n_{t}}\log\left(1+e^{-y_{i}v_{i}}\right),\;\;\partial L\left(v\right)=\left\{x\right\},\;\mathrm{where}\;x_{i}=\frac{-y_{i}e^{-y_{i}v_{i}}}{1+e^{-y_{i}v_{i}}},\;\forall i\in \left\{1\ldots n_{t}\right\}.$$
  First, observe that the loss L(·) satisfies the following inequality:
  (A4) $$\begin{array}{c} \left|L(v)\right|\leq n_{t}+{∥v∥}_{1}. \end{array}$$
  This means that there exists $R_{1}>0$ such that the following inequality is valid:
  (A5) $$\begin{array}{c} \underset{∥v∥\leq C\sqrt{n_{t}}}{\sup}\left|L(v)\right|\leq R_{1}n_{t}. \end{array}$$
  Additionally, the following results hold true:
  (A6) $$\begin{array}{cc} \underset{∥v∥\leq C\sqrt{n_{t}}}{\sup}\;\underset{s\in \partial L(v)}{\sup}∥s∥ & =\underset{∥v∥\leq C\sqrt{n_{t}}}{\sup}∥x∥\leq {\left(\sum_{i=1}^{n_{t}}y_{i}^{2}\right)}^{\frac{1}{2}}. \end{array}$$
  This means that there exists $R_{2}>0$ such that the following inequality is valid:
  (A7) $$\begin{array}{c} \underset{∥v∥\leq C\sqrt{n_{t}}}{\sup}\;\underset{s\in \partial L(v)}{\sup}∥s∥\leq R_{2}\sqrt{n_{t}}. \end{array}$$
  Then, there exists a universal constant R>0 such that Assumption 3 is satisfied for the logistic loss for any λ>0.
3. Hinge loss: Finally, we consider the hinge loss applied to a binary classification model (i.e., $y_{i}\in \left\{-1,1\right\}$). It is clear that the hinge loss is a proper convex function in R. Moreover, L(·) is given by $L\left(\cdot \right)=\sum_{i=1}^{n_{t}}\max\left(1-y_{i}v_{i},0\right)$. Following [33], the sub-differential set ∂L(·) can be expressed as follows:
  (A8) $$\begin{array}{c} \partial L\left(v\right)=\left\{\frac{1}{2}D\left(1+g\right):{∥g∥}_{\infty }\leq 1,g^{⊤}\left(Dv+1\right)={∥Dv+1∥}_{1}\right\}, \end{array}$$
  where $D\in R^{n_{t}\times n_{t}}$ is a diagonal matrix with diagonal entries ${\left\{y_{i}\right\}}_{i=1}^{n_{t}}$. Note that the loss function L(·) satisfies the following inequality:
  (A9) $$\begin{array}{c} \left|L(v)\right|\leq n_{t}+{∥v∥}_{1}. \end{array}$$
  This means that there exists $R_{1}>0$ such that the following inequality is valid:
  (A10) $$\begin{array}{c} \underset{∥v∥\leq C\sqrt{n_{t}}}{\sup}\left|L(v)\right|\leq R_{1}n_{t}. \end{array}$$
  Moreover, the result in (A8) shows that any element s in the sub-differential set ∂L(v) satisfies the following:
  (A11) $$\begin{array}{c} ∥s∥\leq \frac{1}{2}\sqrt{n_{t}}+\frac{1}{2}∥g∥\leq \frac{1}{2}\sqrt{n_{t}}+\frac{1}{2}\sqrt{n_{t}}{∥g∥}_{\infty }\leq \sqrt{n_{t}}. \end{array}$$
  This means that there exists $R_{2}>0$ such that the following inequality is valid:
  (A12) $$\begin{array}{c} \underset{∥v∥\leq C\sqrt{n_{t}}}{\sup}\;\underset{s\in \partial L(v)}{\sup}∥s∥\leq R_{2}\sqrt{n_{t}}. \end{array}$$
  Then, there exists a universal constant R>0 such that Assumption 3 is satisfied for the hinge loss for any λ>0.

## Appendix B. Proof of Lemma 1

### Appendix B.1. Primal Compactness

We start our analysis by assuming that λ>0. We first consider the compactness of the source problem given in (4). Note that the formulation in (4) has a unique optimal solution. Assume that ${\hat{w}}_{s,p}\in R^{p}$ is the unique optimal solution of the optimization problem given in (4). The analysis in [20] (Lemma 1) can be used to prove that there exists $C_{1}>0$ such that the following inequality is valid:

(A13) $$\begin{array}{c} {∥{\hat{w}}_{s,p}∥}^{2}\leq C_{1}, \end{array}$$

with probability going to one as p→∞. Moreover, observe that the formulation in (33) has a unique optimal solution. Assume that ${\hat{w}}_{t,p}\in R^{p}$ is the unique optimal solution of the optimization problem given in (33). Assumption 3 supposes that the loss function is proper. Then, we can conclude that there exists $C_{2}>0$ such that

(A14) $$\begin{array}{c} \ell \left(y,z\right)\geq -C_{2},\;\forall z\in R. \end{array}$$

Now, we define $O_{t,p}^{★}$ as the optimal objective value of the formulation in (33). Then, we can see that there exists $C_{3}>0$ such that

(A15) $$\begin{array}{c} \frac{\lambda }{2}{∥{\hat{w}}_{t,p}∥}^{2}\leq O_{t,p}^{★}+C_{3}. \end{array}$$

Given that ${\hat{w}}_{s,p}$ is a feasible solution in the formulation given in (33), we obtain the following inequality:

(A16) $$\begin{array}{c} \frac{\lambda }{2}{∥{\hat{w}}_{t,p}∥}^{2}\leq \frac{1}{p}\sum_{i=1}^{p}\ell \left(y_{i};a_{i}^{⊤}{\hat{w}}_{s,p}\right)+\frac{\lambda }{2}{∥{\hat{w}}_{s,p}∥}^{2}+C_{3}. \end{array}$$

Based on [27] (Theorem 2.1), the following convergence in probability holds:

(A17) $$\begin{array}{c} \frac{∥A∥}{\sqrt{n_{t}}}\overset{p\to +\infty }{\to }\frac{\sqrt{\alpha_{t}}+1}{\sqrt{\alpha_{t}}}, \end{array}$$

where the matrix $A\in R^{n_{t}\times p}$ is formed by the concatenation of vectors ${\left\{a_{i}\right\}}_{i=1}^{n_{t}}$. Then, there exists $C_{4}>0$ such that the following inequality is valid:

(A18) $$\begin{array}{c} ∥A{\hat{w}}_{s,p}∥\leq ∥A∥∥{\hat{w}}_{s,p}∥\leq C_{4}\sqrt{n_{t}}, \end{array}$$

with probability going to one as p→∞. Combining this with the assumption in (12), we see that there exists $C_{5}>0$ such that the following inequality is valid:

(A19) $$\begin{array}{c} \frac{1}{n_{t}}\left|\sum_{i=1}^{p}\ell \left(y_{i};a_{i}^{⊤}{\hat{w}}_{s,p}\right)\right|\leq C_{5}. \end{array}$$

Given that λ>0 and the result in (A13), we conclude that there exists $C_{6}>0$ such that the following holds:

(A20) $$\begin{array}{c} {∥{\hat{w}}_{t,p}∥}^{2}\leq C_{6}, \end{array}$$

with probability going to one as p→∞.

Now, we consider the case when λ=0. Define $g_{s,p}\left(\cdot \right)$, $O_{s,p}^{★}$, and ${\hat{w}}_{s,p}$ as the cost function, the optimal cost value, and the optimal solution of the formulation in (4). Moreover, define $g_{t,p}\left(\cdot \right)$, $O_{t,p}^{★}$, and ${\hat{w}}_{t,p}$ as the cost function, the optimal cost value, and the optimal solution of the formulation in (33). Note that the loss function ℓ(y,·) is strongly convex with a strong convexity parameter S>0. Then, for any $x_{1},x_{2}\in R$, the following property is valid:

(A21) $$\begin{array}{c} \ell \left(y,\frac{x_{1}+x_{2}}{2}\right)\leq \frac{1}{2}\ell \left(y,x_{1}\right)+\frac{1}{2}\ell \left(y,x_{2}\right)-\frac{S}{8}{\left|x_{1}-x_{2}\right|}^{2}. \end{array}$$

This means that, for any i∈{1,…,n}, the following property is valid:

(A22) $$\begin{array}{c} \ell \left(y_{i},\frac{a_{i}^{⊤}w_{1}+a_{i}^{⊤}w_{2}}{2}\right)\leq \frac{1}{2}\ell \left(y_{i},a_{i}^{⊤}w_{1}\right)+\frac{1}{2}\ell \left(y_{i},a_{i}^{⊤}w_{2}\right)-\frac{S}{8}{\left|a_{i}^{⊤}w_{1}-a_{i}^{⊤}w_{2}\right|}^{2}, \end{array}$$

where *n* can be the number of samples of the source task or target task and ${\left\{y_{i}\right\}}_{i=1}^{n}$ are the labels of the source task or target task. Given the convexity of the norm, we obtain the following inequality:

(A23) $$\begin{array}{c} g_{p}\left(\frac{w_{1}+w_{2}}{2}\right)\leq \frac{1}{2}g_{p}\left(w_{1}\right)+\frac{1}{2}g_{p}\left(w_{2}\right)-\frac{S}{8p}{∥A\left(w_{1}-w_{2}\right)∥}^{2}, \end{array}$$

where $g_{p}\left(\cdot \right)$ can be the cost value of the source task or target task formulations. Now, we focus on the source formulation. Take $w_{1}={\hat{w}}_{s,p}$ and $w_{2}=0$. Moreover, see that the loss function is proper. Then, there exists $C_{7}>0$ such that

(A24) $$\begin{array}{c} \frac{S}{8p}{∥A{\hat{w}}_{s,p}∥}^{2}\leq C_{7}+\frac{1}{2}g_{s,p}\left(0\right). \end{array}$$

Given the assumption in (12), S>0, $\alpha_{s}>1$, and the analysis in [27] (Theorem 2.1), there exists $C_{8}>0$ such that

(A25) $$\begin{array}{c} {∥{\hat{w}}_{s,p}∥}^{2}\leq C_{8}. \end{array}$$

Now, we focus on the target task. Take $w_{1}={\hat{w}}_{t,p}$ and $w_{2}={\hat{w}}_{s,p}$. Moreover, see that the loss function is proper. Then, there exists $C_{9}>0$ such that

(A26) $$\begin{array}{c} \frac{S}{8p}{∥A\left({\hat{w}}_{t,p}-{\hat{w}}_{s,p}\right)∥}^{2}\leq C_{9}+\frac{1}{2}g_{t,p}\left({\hat{w}}_{s,p}\right). \end{array}$$

Given the assumption in (12), the result in (A25), S>0, $\alpha_{t}>1$, and the analysis in [27] (Theorem 2.1), there exists $C_{10}>0$ such that

(A27) $$\begin{array}{c} {∥{\hat{w}}_{t,p}∥}^{2}\leq C_{10}. \end{array}$$

This completes the first part of the proof of Lemma 1.

### Appendix B.2. Dual Compactness

The analysis in Appendix B.1 shows that the formulation in (34) can be equivalently formulated, where the primal feasibility set is given by

(A28) $$\begin{array}{c} {∥w∥}^{2}\leq C, \end{array}$$

where C>0 is a sufficiently large constant that satisfies the analysis in Appendix B.1. Now, define ${\hat{u}}_{p}$ as the optimal solution of the formulation in (34). Additionally, define the function $L^{★}\left(\cdot \right)$ as $L^{★}\left(u\right)=\sum_{i=1}^{n_{t}}\ell^{★}\left(y_{i};u_{i}\right)$. We can see that the optimal vector ${\hat{u}}_{p}$ solves the following maximization problem:

$$\begin{array}{c} {\hat{u}}_{p}=\underset{u\in R^{n_{t}}}{\mathrm{argmax}}\;u^{⊤}Aw-L^{★}\left(u\right), \end{array}$$

where the data matrix $A={\left[a_{1},\ldots ,a_{n_{t}}\right]}^{⊤}\in R^{n_{t}\times p}$. Now, we denote by $\partial L^{★}\left(u\right)$ the sub-differential set of the function $L^{★}\left(\cdot \right)$ evaluated at u. Therefore, the solution of the above maximization problem satisfies the following condition:

(A29) $$\begin{array}{c} Aw\in \partial L^{★}\left({\hat{u}}_{p}\right). \end{array}$$

Now, we use the result in [25] (Proposition 11.3) to show that the condition in (A29) can be equivalently expressed as follows:

(A30) $$\begin{array}{c} {\hat{u}}_{p}\in \partial L\left(Aw\right), \end{array}$$

where the loss function $L\left(w\right)=\sum_{i=1}^{n_{t}}\ell \left(y_{i};w_{i}\right)$ based on [25] (Proposition 11.22). Note that the introduced constraint in (A28) is satisfied. Moreover, the analysis presented in (A17) shows that there exists $C_{1}>0$ such that the following inequality holds:

(A31) $$\begin{array}{c} {∥Aw∥}^{2}\leq C_{1}n_{t}, \end{array}$$

with probability going to one as *p* goes to +∞. Now, we use the assumption in (12) to conclude that there exists $C_{2}>0$ such that the following inequality holds:

(A32) $$\begin{array}{c} {∥{\hat{u}}_{p}∥}^{2}\leq C_{2}n_{t}, \end{array}$$

with probability going to one as *p* goes to +∞. This completes the proof of Lemma 1.

## Appendix C. Proof of Lemma 2

To prove the convergence properties stated in Lemma 2, we show first that they are valid for the auxiliary formulation corresponding to the source problem.

### Appendix C.1. Auxiliary Convergence

Note that the analysis present in Section 7 is also valid for the source problem. This is because the formulation in (8) is equivalent to the source problem in (4) if Σ is the all zero matrix and we use the source training data. Then, we can see that the optimal solution of the auxiliary formulation corresponding to the source problem, denoted by ${\tilde{w}}_{s}$, can be expressed as follows:

(A33) $$\begin{array}{c} {\tilde{w}}_{s}=q_{p,s}^{★}\xi_{s}-\frac{r_{p,s}^{★}}{∥{\tilde{g}}_{s}∥}B_{\xi_{s}}^{⊥}{\tilde{g}}_{s}, \end{array}$$

where ${\tilde{g}}_{s}={\left(B_{\xi_{s}}^{⊥}\right)}^{⊤}g_{s}$ and $g_{s}$ has independent standard Gaussian components. Here, $B_{\xi_{s}}^{⊥}\in R^{p\times (p-1)}$ is formed by an orthonormal basis orthogonal to the vector $\xi_{s}$. Additionally, our analysis in Section 7 shows that the following convergence in probability holds:

(A34) $$\begin{array}{c} q_{p,s}\overset{p\to +\infty }{\to }q_{s}^{★}\;\mathrm{and}\;r_{p,s}^{★}\overset{p\to +\infty }{\to }r_{s}^{★}. \end{array}$$

Here, $q_{s}^{★}$ and $r_{s}^{★}$ are the optimal solutions of the asymptotic limit of the source formulation defined in (14).

Note that $\mu_{p}$ can be expressed as follows:

(A35) $$\begin{array}{c} \mu_{p}=\sigma_{\min}\left({\left(B_{\xi_{t}}^{⊥}\right)}^{⊤}\Lambda B_{\xi_{t}}^{⊥}\right). \end{array}$$

Using the eigenvalue interlacing theorem, one can see that

(A36) $$\begin{array}{c} \sigma_{\min,1}\left(\Lambda \right)\leq \mu_{p}\leq \sigma_{\min,2}\left(\Lambda \right). \end{array}$$

Then, using the assumption in (13), we can see that the random variable $\mu_{p}$ converges in probability to $\mu_{\min}$, where $\mu_{\min}$ is defined in Assumption 5. Now, we study the properties of the remaining functions using the optimal solution of the auxiliary formulation defined in (A33), i.e., ${\tilde{w}}_{s}$, instead of ${\hat{w}}_{s}$. For instance, we first study the random sequence ${\tilde{V}}_{p,ts}=\xi_{t}^{⊤}\Lambda {\tilde{w}}_{s}$ to infer the asymptotic properties of $V_{p,ts}$.

First, fix $\sigma >-\mu_{\min}$. Then, based on the convergence of $\mu_{p}$ and [34] (Proposition 3), the sequence of random functions $T_{p,g}\left(.\right)$ converges in probability as follows:

(A37) $$\begin{array}{c} T_{p,g}\left(\sigma \right)\overset{p\to +\infty }{\to }T_{g}\left(\sigma \right)=E_{\mu }\left[1/\left(\mu +\sigma \right)\right]. \end{array}$$

Now, we express σ as $\sigma =\sigma^{\prime }-x$, where $\sigma^{\prime }>0$. This means that the following convergence in probability holds true:

(A38) $$\begin{array}{c} T_{p,g}\left(\sigma^{\prime }-x\right)\overset{p\to +\infty }{\to }T_{g}\left(\sigma^{\prime }-x\right), \end{array}$$

for any $x<\sigma^{\prime }+\mu_{\min}$. Note that the functions $T_{p,g}\left(.\right)$ and $T_{g}\left(.\right)$ are both convex and continuous in the variable *x* in the set $[0,\;\sigma^{\prime }+\mu_{\min}[$. Then, based on [30] (Theorem II.1), the convergence in (A38) is uniform in the variable *x* in the compact set $\left[0,\;\sigma^{\prime }/2+\mu_{\min}\right]$. Now, note that $\mu_{p}$ converges in probability to $\mu_{\min}$. Therefore, we obtain the following convergence in probability:

(A39) $$\begin{array}{c} T_{p,g}\left(\sigma^{\prime }-\mu_{p}\right)\overset{p\to +\infty }{\to }T_{g}\left(\sigma^{\prime }-\mu_{\min}\right), \end{array}$$

valid for any fixed $\sigma^{\prime }>0$. Using the block matrix inversion lemma, the function $T_{p,t}\left(.\right)$ can be expressed as follows:

(A40) $$\begin{array}{cc} T_{p,t}\left(\sigma \right) & =\xi_{t}^{⊤}\Lambda B_{\xi_{t}}^{⊥}{\left[{\left(B_{\xi_{t}}^{⊥}\right)}^{⊤}\Lambda B_{\xi_{t}}^{⊥}+\sigma I_{p-1}\right]}^{-1}{\left(B_{\xi_{t}}^{⊥}\right)}^{⊤}\Lambda \xi_{t}\\ \; & =\xi_{t}^{⊤}\Lambda \xi_{t}+\sigma -\frac{1}{\xi_{t}^{⊤}{\left[\Lambda +\sigma I_{p}\right]}^{-1}\xi_{t}}. \end{array}$$

Therefore, we obtain the following expression:

(A41) $$\begin{array}{c} Z_{p,t}\left(\sigma \right)=\sigma -\frac{1}{\xi_{t}^{⊤}{\left[\Lambda +\sigma I_{p}\right]}^{-1}\xi_{t}}. \end{array}$$

Then, using the theoretical results stated in [34] (Proposition 3), the functions $Z_{p,t}\left(.\right)$ converges in probability as follows:

(A42) $$\begin{array}{c} Z_{p,t}\left(\sigma \right)\overset{p\to +\infty }{\to }Z_{t}\left(\sigma \right)=\sigma -\frac{1}{E_{\mu }\left[1/\left(\mu +\sigma \right)\right]}. \end{array}$$

Combine this with the above analysis to obtain the following convergence in probability:

(A43) $$\begin{array}{c} Z_{p,t}\left(\sigma^{\prime }-\mu_{p}\right)\overset{p\to +\infty }{\to }Z_{t}\left(\sigma^{\prime }-\mu_{\min}\right), \end{array}$$

valid for any $\sigma^{\prime }>0$. Based on the result in (A33), the sequence of random functions ${\tilde{Z}}_{p,ts}\left(.\right)$ converges in probability to the following function:

(A44) $$\begin{array}{c} Z_{ts}\left(\sigma \right)=q_{s}^{★}\rho Z_{t}\left(\sigma \right). \end{array}$$

Combine this with the above analysis to obtain the following convergence in probability:

(A45) $$\begin{array}{c} {\tilde{Z}}_{p,ts}\left(\sigma^{\prime }-\mu_{p}\right)\overset{p\to +\infty }{\to }Z_{ts}\left(\sigma^{\prime }-\mu_{\min}\right), \end{array}$$

valid for any $\sigma^{\prime }>0$. Using the same analysis and based on (A33) and (A34), one can see that the sequence of random functions ${\tilde{Z}}_{p,s}\left(.\right)$ converges in probability to the following function:

(A46) $$\begin{array}{cc} {\tilde{Z}}_{p,s}\left(\sigma \right) & \overset{p\to +\infty }{\to }Z_{s}\left(\sigma \right)={\left(\rho q_{s}^{★}\right)}^{2}Z_{t}\left(\sigma \right)\\ \; & -\left(\left(1-\rho^{2}\right){\left(q_{s}^{★}\right)}^{2}+{\left(r_{s}^{★}\right)}^{2}\right)E_{\mu }\left[\mu \sigma /(\mu +\sigma )\right]. \end{array}$$

Combine this with the above analysis to obtain the following convergence in probability:

(A47) $$\begin{array}{c} {\tilde{Z}}_{p,s}\left(\sigma^{\prime }-\mu_{p}\right)\overset{p\to +\infty }{\to }Z_{s}\left(\sigma^{\prime }-\mu_{\min}\right), \end{array}$$

valid for any $\sigma^{\prime }>0$. The above analysis shows that the asymptotic properties stated in Lemma 2 are valid for the AO formulation corresponding to the source problem. Now, it remains to show that these properties also hold for the primary formulation.

### Appendix C.2. Primary Convergence

Here, we assume that λ>0. The case when λ=0 can be conducted similarly. Now, we show that the convergence properties proved above are also valid for the primary problem. To this end, we show that all the assumptions in Theorem 2 are satisfied. We start our proof by defining the following open set:

$$\begin{array}{c} T_{\epsilon }=\{w\in R^{p}:|\xi_{t}^{⊤}{\left[\Lambda +\sigma I_{p}\right]}^{-1}w-\rho q_{s}^{★}K\left|<\epsilon \right\}, \end{array}$$

where *K* is defined as follows:

(A48) $$\begin{array}{c} K=E_{\mu }\left[1/\left(\mu +\sigma \right)\right]. \end{array}$$

Now, we consider the feasibility set $D_{\epsilon }=T_{1}/S_{\epsilon }$, where $T_{1}$ is defined in (41). Based on the analysis of the generalized target formulation in Section 7.3.2, one can see that the AO formulation corresponding to the source formulation with the set $D_{\epsilon }$ can be asymptotically expressed as follows:

$$\begin{array}{cc} \; & V_{p}:\min_{\begin{array}{c} \left(q_{s},r_{s}\right)\in T_{2} \end{array}}\min_{\begin{array}{c} r_{s}\in {\tilde{D}}_{\epsilon } \end{array}}\max_{u\in C_{s}}\frac{∥u∥}{p}g_{s}^{⊤}B_{\xi_{s}}^{⊥}r_{s}+\frac{q_{s}}{p}u^{⊤}s_{s}\\ \; & +\frac{\lambda }{2}\left(q_{s}^{2}+{∥r_{s}∥}^{2}\right)+\frac{1}{p}∥r_{s}∥h_{s}^{⊤}u-\frac{1}{p}\sum_{i=1}^{n_{s}}\ell^{★}\left(y_{s,i};u_{i}\right). \end{array}$$

Here, the feasibility set $T_{2}$ is defined in Section 7.3.2 and the feasibility set ${\tilde{D}}_{\epsilon }$ is given by

$$\begin{array}{cc} \; & \{r_{s}:\left|q_{s}\rho K_{p,t}+q_{s}\sqrt{1-\rho^{2}}K_{p,r}+\xi_{t}^{⊤}{\left[\Lambda +\sigma I_{p}\right]}^{-1}B_{\xi_{s}}^{⊥}r_{s}-\rho q_{s}^{★}K\right|\geq \epsilon \\ \; & \;\;\;\;\;\;\;,∥r_{s}∥=r_{s}\}. \end{array}$$

This follows based on the decomposition in (40) and where $K_{p,t}=\xi_{t}^{⊤}{\left[\Lambda +\sigma I_{p}\right]}^{-1}\xi_{t}$ and $K_{p,r}=\xi_{t}^{⊤}{\left[\Lambda +\sigma I_{p}\right]}^{-1}\xi_{r}$. Note that the optimization problem given in $V_{p}$ can be equivalently formulated as follows:

$$\begin{array}{cc} \; & V_{p}:\min_{\begin{array}{c} \left(q_{s},r_{s}\right)\in {\hat{S}}_{\epsilon } \end{array}}\min_{\begin{array}{c} r_{s}\in {\tilde{D}}_{\epsilon } \end{array}}\max_{u\in C_{s}}\frac{∥u∥}{p}g_{s}^{⊤}B_{\xi }^{⊥}r_{s}+\frac{q_{s}}{p}u^{⊤}s_{s}\\ \; & +\frac{\lambda }{2}\left(q_{s}^{2}+{∥r_{s}∥}^{2}\right)+\frac{1}{p}∥r_{s}∥h_{s}^{⊤}u-\frac{1}{p}\sum_{i=1}^{n_{s}}\ell^{★}\left(y_{s,i};u_{i}\right). \end{array}$$

Here, we replace the feasibility set $T_{2}$ by the feasibility set ${\hat{S}}_{\epsilon }$ defined as follows:

$$\begin{array}{cc} \; & \left\{\right|q_{s}\rho K_{p,t}+q_{s}\sqrt{1-\rho^{2}}K_{p,r}-r_{s}\xi_{t}^{⊤}{\left[\Lambda +\sigma I_{p}\right]}^{-1}B_{\xi_{s}}^{⊥}\frac{{\tilde{g}}_{s}}{∥{\tilde{g}}_{s}∥}-\rho q_{s}^{★}K|\\ \; & \;\;\;\;\;\geq \epsilon \}\cap T_{2}, \end{array}$$

where ${\tilde{g}}_{s}={\left(B_{\xi_{s}}^{⊥}\right)}^{⊤}g_{s}$. This follows since the first set in ${\hat{S}}_{\epsilon }$ satisfies the condition in the set ${\tilde{D}}_{\epsilon }$. Now, assume that ${\hat{\phi }}_{p}^{★}$ is the optimal cost value of the optimization problem $V_{p}$ and define the function ${\hat{h}}_{p}\left(.\right)$ as follows:

$$\begin{array}{cc} \; & {\hat{h}}_{p}\left(q_{s},r_{s}\right)=\min_{\begin{array}{c} r_{s}\in {\tilde{D}}_{\epsilon } \end{array}}\max_{u\in C_{s}}\frac{∥u∥}{p}g_{s}^{⊤}B_{\xi_{s}}^{⊥}r_{s}+\frac{q_{s}u^{⊤}s_{s}}{p}\\ \; & +\frac{\lambda }{2}\left(q_{s}^{2}+r_{s}^{2}\right)+\frac{r_{s}}{p}h_{s}^{⊤}u-\frac{1}{p}\sum_{i=1}^{n_{s}}\ell^{★}\left(y_{s,i};u_{i}\right), \end{array}$$

in the set ${\hat{S}}_{\epsilon }$. Based on the max–min inequality [35], the function ${\hat{h}}_{p}\left(.\right)$ can be lower bounded by the following function:

$$\begin{array}{cc} \; & {\tilde{h}}_{p}\left(q_{s},r_{s}\right)=\max_{u\in C_{s}}\min_{\begin{array}{c} r_{s}\in {\tilde{D}}_{\epsilon } \end{array}}\frac{∥u∥}{p}g_{s}^{⊤}B_{\xi_{s}}^{⊥}r_{s}+\frac{q_{s}u^{⊤}s_{s}}{p}\\ \; & +\frac{\lambda }{2}\left(q_{s}^{2}+r_{s}^{2}\right)+\frac{r_{s}}{p}h_{s}^{⊤}u-\frac{1}{p}\sum_{i=1}^{n_{s}}\ell^{★}\left(y_{s,i};u_{i}\right). \end{array}$$

This is valid for any $\left(q_{s},r_{s}\right)\in {\hat{S}}_{\epsilon }$. Moreover, note that the following inequality holds true:

(A49) $$\begin{array}{c} \min_{\begin{array}{c} r_{s}\in {\tilde{D}}_{\epsilon } \end{array}}\frac{∥u∥}{p}g_{s}^{⊤}B_{\xi_{s}}^{⊥}r_{s}\geq -\frac{∥u∥}{p}∥{\left(B_{\xi_{s}}^{⊥}\right)}^{⊤}g_{s}∥r_{s}, \end{array}$$

for any $\left(q_{s},r_{s}\right)\in {\hat{S}}_{\epsilon }$. Following the generalized analysis in Section 7.3.2, one can see that the auxiliary problem corresponding to the source formulation can be expressed as follows:

(A50) $$\begin{array}{cc} \; & \min_{\begin{array}{c} \left(q_{s},r_{s}\right)\in T_{2} \end{array}}\underset{\sigma_{s}>0}{\sup}\frac{1}{n_{s}}\sum_{i=1}^{n_{s}}M_{\ell (y_{s,i},.)}\left(r_{s}h_{s,i}+q_{s}s_{s,i};\frac{r_{s}∥{\tilde{g}}_{s}∥}{\sqrt{n_{s}}\sigma_{s}}\right)\\ \; & -\frac{r_{s}\sigma_{s}}{2}\frac{∥{\tilde{g}}_{s}∥}{\sqrt{n_{s}}}+\frac{\lambda }{2}\left(q_{s}^{2}+r_{s}^{2}\right). \end{array}$$

This means that the function ${\tilde{h}}_{p}\left(.\right)$ can be lower bounded by the cost function of the minimization problem formulated in (A50) denoted by ${\hat{g}}_{p}\left(.\right)$, i.e.,

(A51) $$\begin{array}{c} {\hat{g}}_{p}\left(q_{s},r_{s}\right)\leq {\tilde{h}}_{p}\left(q_{s},r_{s}\right). \end{array}$$

Here, both functions are defined in the feasibility set ${\hat{S}}_{\epsilon }$. Now, define $\phi_{p}^{★}$ as the optimal cost value of the auxiliary optimization problem corresponding to the source formulation defined in Section 7.3.1. Note that the loss function ${\hat{g}}_{p}\left(.\right)$ is strongly convex in the variables $\left(q_{s},r_{s}\right)$ with strong convexity parameter λ>0. This means that, for any β∈[0,1], $\left(q_{s,1},r_{s,1}\right)\in T_{2}$ and $\left(q_{s,2},r_{s,2}\right)\in T_{2}$, we have the following inequality:

(A52) $$\begin{array}{cc} \; & {\hat{g}}_{p}\left(\beta v_{1}+\left(1-\beta \right)v_{2}\right)\leq \beta {\hat{g}}_{p}\left(v_{1}\right)\\ \; & +\left(1-\beta \right){\hat{g}}_{p}\left(v_{2}\right)-\frac{\lambda }{2}\beta \left(1-\beta \right){∥v_{1}-v_{2}∥}^{2}, \end{array}$$

where $v_{1}=\left[q_{s,1},r_{s,1}\right]$ and $v_{2}=\left[q_{s,2},r_{s,2}\right]$. Take $v_{1}$ as $v_{p}^{★}$, which represents the optimal solution of the optimization problem (A50). Then, the inequality in (A52) implies the following inequality:

(A53) $$\begin{array}{cc} \; & \phi_{p}^{★}\leq {\hat{g}}_{p}\left(v_{2}\right)-\frac{\lambda }{2}\beta {∥v_{p}^{★}-v_{2}∥}^{2}. \end{array}$$

This is valid for any $v_{2}$ in the set $T_{2}$. Now, taking β=1/2 and the minimum over $v_{2}$ in the set ${\hat{S}}_{\epsilon }$ in both sides, we obtain the following inequality:

$$\begin{array}{c} \phi_{p}^{★}+\frac{\lambda }{4}\min_{v\in {\hat{S}}_{\epsilon }}{∥v_{p}^{★}-v∥}^{2}\leq \min_{v\in {\hat{S}}_{\epsilon }}{\hat{g}}_{p}\left(v\right). \end{array}$$

Based on the above analysis, note that the following inequality also holds true:

(A54) $$\begin{array}{c} \min_{v\in {\hat{S}}_{\epsilon }}{\hat{g}}_{p}\left(v\right)\leq {\hat{\phi }}_{p}^{★}. \end{array}$$

Then, to verify the assumption of [17] (Theorem 6.1), it remains to show that there exists $\epsilon^{\prime }>0$ such that, the following inequality holds:

(A55) $$\begin{array}{c} \frac{\lambda }{4}\min_{v\in {\hat{S}}_{\epsilon }}{∥v_{p}^{★}-v∥}^{2}\geq \epsilon^{\prime }, \end{array}$$

with probability going to 1 as p→∞. Note that any element in the set ${\hat{S}}_{\epsilon }$ satisfies the following inequality:

$$\begin{array}{cc} \; & \epsilon \leq |q_{s}\rho K_{p,t}+q_{s}\sqrt{1-\rho^{2}}K_{p,r}-r_{s}\xi_{t}^{⊤}{\left[\Lambda +\sigma I_{p}\right]}^{-1}B_{\xi }^{⊥}\frac{{\tilde{g}}_{s}}{∥{\tilde{g}}_{s}∥}-\rho q_{s}^{★}K|\leq \\ \; & |q_{s}\rho K_{p,t}-\rho q_{s}^{★}K\left|+\right|q_{s}\sqrt{1-\rho^{2}}\left|\right|K_{p,r}\left|+\right|r_{s}\left|\right|\xi_{t}^{⊤}{\left[\Lambda +\sigma I_{p}\right]}^{-1}B_{\xi }^{⊥}\frac{{\tilde{g}}_{s}}{∥{\tilde{g}}_{s}∥}|. \end{array}$$

Based on the analysis in Appendix C.1, we have the following convergence in probability:

(A56) $$\begin{array}{c} |q_{s}\rho K_{p,t}-\rho q_{s}^{★}K|\overset{p\to +\infty }{\to }\left|q_{s}-q_{s}^{★}\right|\rho K\\ |q_{s}|\sqrt{1-\rho^{2}}|K_{p,r}|\overset{p\to +\infty }{\to }0,\;|r_{s}\left|\right|\xi_{t}^{⊤}{\left[\Lambda +\sigma I_{p}\right]}^{-1}B_{\xi }^{⊥}\frac{{\tilde{g}}_{s}}{∥{\tilde{g}}_{s}∥}|\overset{p\to +\infty }{\to }0. \end{array}$$

This means that there exists $\epsilon^{\prime \prime }>0$ such that any elements in the set ${\hat{S}}_{\epsilon }$ satisfies the following inequality:

(A57) $$\begin{array}{c} |q_{s}-q_{s}^{★}|\rho K\geq \epsilon^{\prime \prime }, \end{array}$$

with probability going to 1 as p→∞. Combining this with Assumption 5 and the consistency result stated in (A34) shows that there exists $\epsilon^{\prime }>0$ such that the following inequality holds:

(A58) $$\begin{array}{c} \frac{\lambda }{4}\min_{v\in {\hat{D}}_{\epsilon }}{∥v_{p}^{★}-v∥}^{2}\geq \epsilon^{\prime }, \end{array}$$

with probability going to 1 as p→∞. This also proves that there exists $\epsilon^{\prime }>0$ such that the following inequality holds:

(A59) $$\begin{array}{c} {\hat{\phi }}_{p}^{★}\geq \phi_{p}^{★}+\epsilon^{\prime }, \end{array}$$

with probability going to 1 as p→∞. This completes the verification of the assumptions in Theorem 2. This means that the optimal solution of the primary problem belongs to the set $S_{\epsilon }$ on events with probability going to 1 as p→∞. Since the choice of ϵ is arbitrary, we obtain the following asymptotic result:

(A60) $$\begin{array}{c} \xi_{t}^{⊤}{\left[\Lambda +\sigma I_{p}\right]}^{-1}{\hat{w}}_{s}\overset{p\to +\infty }{\to }q_{s}^{★}\rho K, \end{array}$$

where ${\hat{w}}_{s}$ is the optimal solution of the source problem (4). Following the same analysis, one can also show the convergence properties stated in Lemma 2.

## Appendix D. Proof of Lemma 4

Here, we assume that λ>0. The case when λ=0 can be conducted similarly. The cost function of the optimization problem (49) can be expressed as follows:

(A61) $$\begin{array}{cc} O_{t}\left(q_{t},r_{t},\sigma_{t}\right) & =\frac{\lambda }{2}\left(q_{t}^{2}+r_{t}^{2}\right)-\frac{\sigma_{t}r_{t}^{2}}{2}-\frac{1}{2}Z_{s}\left(\sigma_{t}\right)-\frac{1}{2}q_{t}^{2}Z_{t}\left(\sigma_{t}\right)\\ \; & +\alpha_{t}E\left[M_{\ell (Y_{t},.)}\left(r_{t}H_{t}+q_{t}S_{t};T_{g}\left(\sigma_{t}\right)\right)\right]+q_{t}Z_{ts}\left(\sigma_{t}\right). \end{array}$$

Note that the function $O_{t}\left(\cdot ,\cdot ,\cdot \right)$ can be expressed as follows:

(A62) $$\begin{array}{cc} O_{t}\left(q_{t},r_{t},\sigma_{t}\right) & =-\frac{\sigma_{t}r_{t}^{2}}{2}+\frac{1}{2}\left(\left(1-\rho^{2}\right){\left(q_{s}^{★}\right)}^{2}+{\left(r_{s}^{★}\right)}^{2}\right)T_{2}\left(\sigma_{t}\right)\\ \; & \;+\alpha_{t}E\left[M_{\ell (Y_{t},.)}\left(r_{t}H_{t}+q_{t}S_{t};T_{1}\left(\sigma_{t}\right)\right)\right]+\frac{\lambda }{2}\left(q_{t}^{2}+r_{t}^{2}\right)\\ \; & \;-\frac{1}{2}{\left(q_{t}-\rho q_{s}^{★}\right)}^{2}\left(\sigma_{t}-1/T_{1}\left(\sigma_{t}\right)\right). \end{array}$$

Here, the functions $T_{1}\left(.\right)$ and $T_{2}\left(.\right)$ are defined as follows:

$$\begin{array}{c} T_{1}\left(\sigma_{t}\right)=E_{\mu }\left[1/\left(\mu +\sigma_{t}\right)\right],\;T_{2}\left(\sigma_{t}\right)=E_{\mu }\left[\mu \sigma_{t}/\left(\mu +\sigma_{t}\right)\right]. \end{array}$$

Based on Assumption 5, the functions $T_{1}\left(\cdot \right)$ and $T_{2}\left(\cdot \right)$ are twice continuously differentiable in the feasibility set. We start our analysis by showing that the function $O_{t}\left(\cdot ,\cdot ,\cdot \right)$ is concave in the variable $\sigma_{t}$ for fixed feasible $\left(q_{t},r_{t}\right)$. First, note that the function $T_{2}\left(\cdot \right)$ is concave in the feasibility set. Now, define the function g(·) as follows:

(A63) $$\begin{array}{c} g\left(\sigma_{t}\right)=\frac{1}{T_{1}\left(\sigma_{t}\right)}. \end{array}$$

Then, we can see that the second derivative of the function g(·) can be expressed as follows:

(A64) $$\begin{array}{c} g^{\prime \prime }\left(\sigma_{t}\right)=-\frac{T_{1}^{\prime \prime }\left(\sigma_{t}\right)T_{1}\left(\sigma_{t}\right)-2T_{1}^{\prime }{\left(\sigma_{t}\right)}^{2}}{T_{1}{\left(\sigma_{t}\right)}^{3}}. \end{array}$$

Here, the first and second derivatives of the function $T_{1}\left(\cdot \right)$ can be expressed as follows:

(A65) $$\begin{array}{c} T_{1}^{\prime }\left(\sigma_{t}\right)=-E_{\mu }\left[1/{\left(\mu +\sigma_{t}\right)}^{2}\right],\;T_{1}^{\prime \prime }\left(\sigma_{t}\right)=2E_{\mu }\left[1/{\left(\mu +\sigma_{t}\right)}^{3}\right]. \end{array}$$

Then, using the Cauchy–Schwarz inequality, one can see that the second derivative of the function g(·) is negative. This implies the concavity of the function g(·). Therefore, using the properties in [35] (Section 3.2), the function $O_{t}\left(\cdot ,\cdot ,\cdot \right)$ is concave in the variable $\sigma_{t}$.

Now, we focus on proving the strong convexity properties. Define the function $O_{t}\left(\cdot ,\cdot \right)$ as follows:

(A66) $$\begin{array}{c} O_{t}\left(q_{t},r_{t}\right)=\underset{\sigma_{t}>-\mu_{\min}}{\sup}O_{t}\left(q_{t},r_{t},\sigma_{t}\right). \end{array}$$

Note that the term $\frac{\lambda }{2}\left(q_{t}^{2}+r_{t}^{2}\right)$ is strongly convex in the variables $\left(q_{t},r_{t}\right)$. Then, to prove our property it suffices to show that the following function is jointly convex in the variables $\left(q_{t},r_{t}\right)$ in the feasibility set:

(A67) $$\begin{array}{cc} h(q_{t},r_{t}) & =\underset{\sigma_{t}>-\mu_{\min}}{\sup}-\frac{\sigma_{t}r_{t}^{2}}{2}+\frac{1}{2}\left(\left(1-\rho^{2}\right){\left(q_{s}^{★}\right)}^{2}+{\left(r_{s}^{★}\right)}^{2}\right)T_{2}\left(\sigma_{t}\right)-\frac{1}{2}{\left(q_{t}-\rho q_{s}^{★}\right)}^{2}\left(\sigma_{t}-1/T_{1}\left(\sigma_{t}\right)\right)\\ \; & \;+\alpha_{t}E\left[M_{\ell (Y_{t},.)}\left(r_{t}H_{t}+q_{t}S_{t};T_{1}\left(\sigma_{t}\right)\right)\right], \end{array}$$

Note that the function h(·,·) can also be expressed as follows:

(A68) $$\begin{array}{cc} h(q_{t},r_{t}) & =\underset{\sigma_{t}>-\mu_{\min}}{\sup}\min_{0\leq \tau \leq C_{\tau }}\frac{\sigma_{t}\tau^{2}}{2}-\tau r_{t}\sigma_{t}+\frac{1}{2}\left(\left(1-\rho^{2}\right){\left(q_{s}^{★}\right)}^{2}+{\left(r_{s}^{★}\right)}^{2}\right)T_{2}\left(\sigma_{t}\right)\\ \; & +\alpha_{t}E\left[M_{\ell (Y_{t},.)}\left(r_{t}H_{t}+q_{t}S_{t};T_{1}\left(\sigma_{t}\right)\right)\right]-\frac{1}{2}{\left(q_{t}-\rho q_{s}^{★}\right)}^{2}\left(\sigma_{t}-1/T_{1}\left(\sigma_{t}\right)\right). \end{array}$$

Here, the feasibility set of the variable τ is bounded given that the optimal τ satisfies $\tau^{★}=r_{t}$. It can be easily seen that the cost function of the optimization problem in (A68) is convex in τ and concave in $\sigma_{t}$. Then, using the result in [36], the function h(·,·) can also be expressed as follows:

(A69) $$\begin{array}{cc} \; & h\left(q_{t},r_{t}\right)=\underset{0<\tau \leq C_{\tau }}{\inf}\underset{\sigma_{t}>-\mu_{\min}}{\sup}\frac{\sigma_{t}\tau }{2}-r_{t}\sigma_{t}+\frac{1}{2}\left(\left(1-\rho^{2}\right){\left(q_{s}^{★}\right)}^{2}+{\left(r_{s}^{★}\right)}^{2}\right)T_{2}\left(\sigma_{t}/\tau \right)\\ \; & +\alpha_{t}E\left[M_{\ell (Y_{t},.)}\left(r_{t}H_{t}+q_{t}S_{t};T_{1}\left(\sigma_{t}/\tau \right)\right)\right]-\frac{1}{2}{\left(q_{t}-\rho q_{s}^{★}\right)}^{2}\left(\sigma_{t}/\tau -1/T_{1}\left(\sigma_{t}/\tau \right)\right). \end{array}$$

Then, to prove our property, it suffices to show that the cost function of the above problem is jointly convex in the variables $\left(q_{t},r_{t},\tau \right)$. Using the positivity of the second derivative, it is easy to see that the function $\tau \to T_{2}\left(\sigma_{t}/\tau \right)$ is convex. Now, using the analysis below Equation (161) in [20] (Appendix H), we can see that the remaining functions are jointly convex in the variables $\left(q_{t},r_{t},\tau \right)$. We omit these steps since they are similar to the approach employed in [20] (Appendix H). This shows that the function $O_{t}\left(\cdot ,\cdot \right)$ is strongly convex in the variables $\left(q_{t},r_{t}\right)$.
